# Transfer activation of SXT/R391 integrative and conjugative elements: unraveling the SetCD regulon

**DOI:** 10.1093/nar/gkv071

**Published:** 2015-02-06

**Authors:** Dominic Poulin-Laprade, Dominick Matteau, Pierre-Étienne Jacques, Sébastien Rodrigue, Vincent Burrus

**Affiliations:** 1 Laboratory of Bacterial Molecular Genetics, Département de biologie, Université de Sherbrooke, Sherbrooke, QC, J1K 2R1, Canada; 2 Laboratory of Microbial Systems and Synthetic Biology, Département de biologie, Université de Sherbrooke, Sherbrooke, QC, J1K 2R1, Canada; 3 Laboratory of Bioinformatics and Genomics, Département de biologie, Université de Sherbrooke, Sherbrooke, QC, J1K 2R1, Canada

## Abstract

Integrative and conjugative elements (ICEs) of the SXT/R391 family have been recognized as key drivers of antibiotic resistance dissemination in the seventh-pandemic lineage of *Vibrio cholerae*. SXT/R391 ICEs propagate by conjugation and integrate site-specifically into the chromosome of a wide range of environmental and clinical *Gammaproteobacteria*. SXT/R391 ICEs bear *setC* and *setD*, two conserved genes coding for a transcriptional activator complex that is essential for activation of conjugative transfer. We used chromatin immunoprecipitation coupled with exonuclease digestion (ChIP-exo) and RNA sequencing (RNA-seq) to characterize the SetCD regulon of three representative members of the SXT/R391 family. We also identified the DNA sequences bound by SetCD in MGI*Vfl*Ind1, a mobilizable genomic island phylogenetically unrelated to SXT/R391 ICEs that hijacks the conjugative machinery of these ICEs to drive its own transfer. SetCD was found to bind a 19-bp sequence that is consistently located near the promoter −35 element of SetCD-activated genes, a position typical of class II transcriptional activators. Furthermore, we refined our understanding of the regulation of excision from and integration into the chromosome for SXT/R391 ICEs and demonstrated that *de novo* expression of SetCD is crucial to allow integration of the incoming ICE DNA into a naive host following conjugative transfer.

## INTRODUCTION

**Figure 1.**
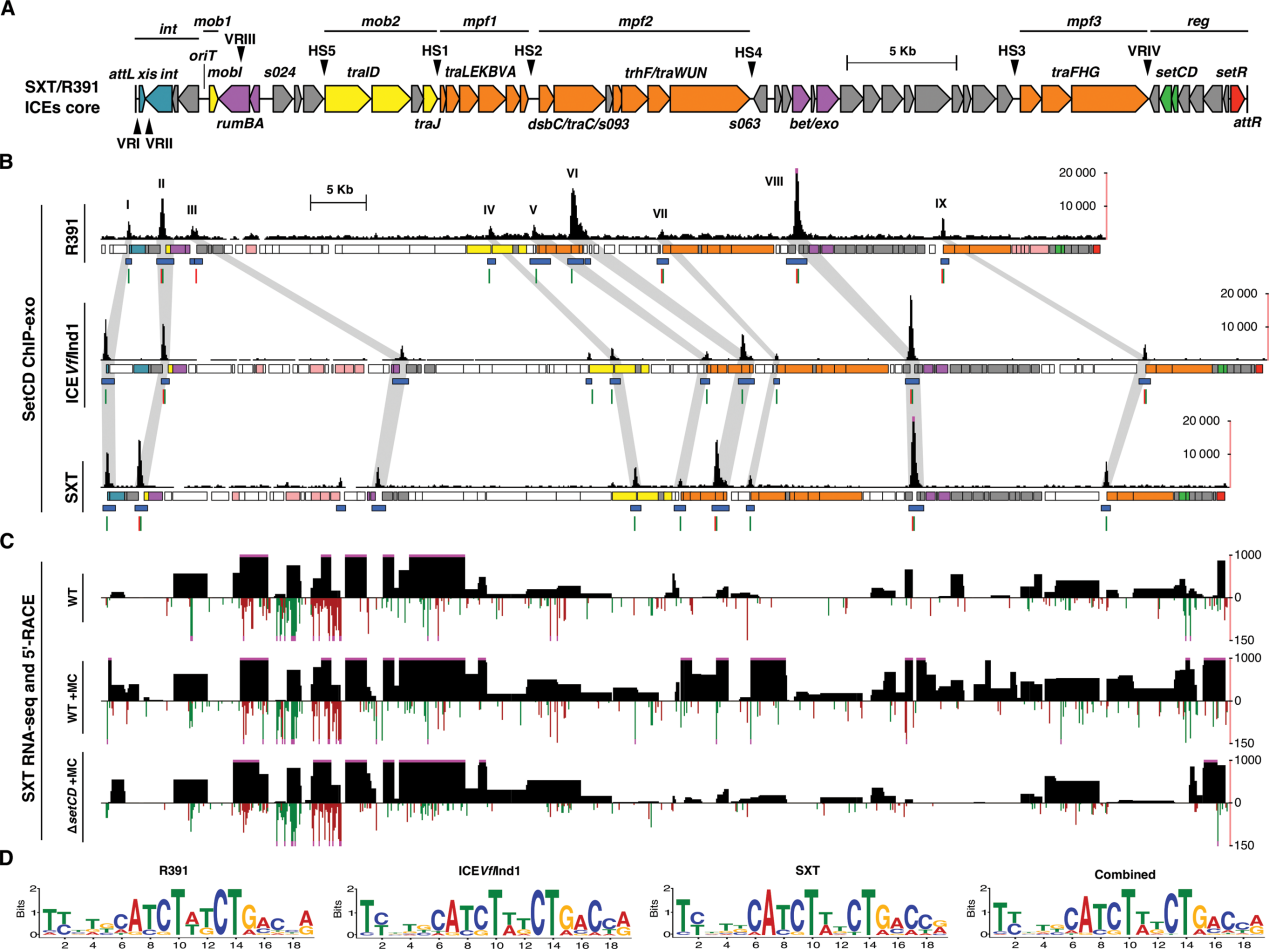
*In vivo* identification of SetCD targets. (**A**) Schematic representation of the conserved genes of SXT/R391 ICEs adapted from Wozniak *et al*. (6). Genes are represented by arrows and are color coded as follow: blue, integration and excision; yellow, DNA processing; orange, mating pair formation; purple, RecA-independent homologous recombination and Umu-like mutagenic repair; green, transcriptional activator; red, transcriptional repressor; grey, other or hypothetical functions. Variable cargo DNA inserted in the conserved core of SXT/R391 ICEs is marked by arrowheads (HS1 to 5 and VRI to IV). The left and right chromosomal attachment sites *attL* and *attR* are also shown. (**B**) SetCD ChIP-exo analysis for R391, ICE*Vfl*Ind1 and SXT. For each ICE, four tracks are shown. First track: number of ChIP-exo reads mapped on ICE DNA sequence (pink dots at the top of black bars indicate a signal beyond the represented y-axis maximal value). Roman numbers indicate ChIP-exo peaks conserved between the three ICEs. Second track: genes from conserved core (same color code as in panel A), variable DNA regions (white) and antibiotic/heavy-metal resistance genes (pink). Third and fourth tracks: position of ChIP-exo enrichment peaks and the position of SetCD-binding motifs, respectively. SetCD motifs were identified for each ICE using the corresponding logo shown in panel D and are represented by green and red tick marks on positive and negative strands, respectively. (**C**) RNA-seq experiments on wild-type SXT in the presence or absence of mitomycin C, and SXT Δ*setCD* in the presence of mitomycin C. For each condition, the upper track shows the reads per kilobase of transcript per million mapped reads value (RPKM) for each gene (black boxes) and the lower track shows the genome-wide 5′-RACE signals (positive strand in green, negative strand in red). Pink dots are as in B. (**D**) Logo sequences recognized by SetCD in R391, ICE*Vfl*Ind1, SXT, as well as the consensus logo of all three ICEs.

Integrative and conjugative elements (ICEs) have recently been shown to be the most abundant conjugative elements in practically all prokaryotic clades (1,2). As such, ICEs are a major driving force of bacterial genome evolution allowing rapid acquisition of a variety of new traits and adaptive functions such as virulence, metabolic pathways and resistance to antimicrobial compounds, heavy metals or bacteriophage infection (3–5). For instance, ICEs of the SXT/R391 family largely contribute to the spread of antibiotic resistance genes in the seventh-pandemic lineage of *Vibrio cholerae*, the etiologic agent of cholera, which remains a major cause of mortality and morbidity on a global scale (6–9). Most SXT/R391 ICEs found in *V. cholerae* clinical isolates confer resistance to sulfamethoxazole and trimethoprim, two antibiotics commonly used for the treatment of cholera (10,11). Since the early 90's, SXT/R391 ICEs have become widespread in environmental and clinical *V. cholerae* isolates from Asia and Africa (7,8). SXT/R391 ICEs are also present in all isolates recovered from cholera patients in Haiti (12–15), are naturally occurring in many other *Gammaproteobacteria* (6,16–18) and are easily transferred to *Escherichia coli* in the laboratory (19). The SXT/R391 ICEs are grouped together because they share a common set of 52 highly conserved genes, among which ∼25 are important for their maintenance, dissemination by conjugation, and regulation (6,20). Highly conserved genes in SXT/R391 ICEs are distributed in seven distinct clusters separated by variable cargo DNA (HS1 to 5 and VRI to IV) (Figure 1A). These conserved clusters consist of *int* (integration/excision), *mob1–2* (DNA processing), *mpf1–3* (mating pair formation modules 1, 2 and 3) and *reg* (regulation) (Figure 1A) (6).

Mobilizable genomic islands (MGIs) are small (<33 kb) genomic islands found in several species of marine *Gammaproteobacteria* (21). SXT/R391 ICEs can mediate the transfer in *trans* of a particular class of MGIs at high frequency by an unusual mechanism. ICE-encoded relaxosome proteins recognize a *cis*-acting locus in MGIs that mimics the origin of transfer (*oriT*) of SXT/R391 ICEs (22). Unlike conjugative plasmids, ICEs and MGIs do not stably maintain by extra-chromosomal replication and must integrate into the host cell's chromosome to be vertically inherited (3,22–23). The gene pairs *int/xis* and *int*_MGI_/*rdfM* are key components for the maintenance of SXT/R391 ICEs and MGIs, respectively. *int* and *int*_MGI_ code for two distinct and unrelated integrases, which mediate the integration of SXT/R391 ICEs into the 5′ end of *prfC* (peptide chain release factor RF3), and the integration of MGIs into the 3′ end of *yicC* (protein of unknown function), respectively (22,24). *xis* and *rdfM* encode recombination directionality factors (RDFs), which facilitate the integrase-mediated excision from the chromosome of SXT/R391 ICEs and MGIs as circular molecules that serve as substrates for the conjugative transfer machinery (23,25).

The conjugative transfer of SXT/R391 ICEs is regulated by three conserved genes located near the *attR* attachment site (Figure 1A) (20). *setR* encodes a λ cI-related transcriptional repressor, which prevents the expression of *setC* and *setD* (26,27). Agents that damage DNA and induce the host SOS response (UV light, mitomycin C, ciprofloxacin) are thought to induce the RecA*-stimulated autoproteolysis and inactivation of SetR, thereby alleviating the repression of *setCD* and allowing excision and transfer of SXT/R391 ICEs (26) (Supplementary Figure S1A). The proteins SetC and SetD are thought to assemble as a heteromeric complex that activates the expression of SXT/R391 genes important for conjugative transfer (20,25). SetCD has also been reported to activate the expression of the *mosAT* toxin-antitoxin system carried by SXT (28) as well as the expression of *int*_MGI_ and *rdfM* of MGIs, thereby triggering the excision of MGIs from the chromosome (22,23). The number of promoters activated by SetCD in SXT/R391 ICEs and the nature of the SetCD operator sites are currently unknown.

In this study we characterized the SetCD regulon and DNA motifs bound by SetCD in three SXT/R391 ICEs and one MGI originating from three different pathogens using chromatin immunoprecipitation coupled to exonuclease digestion (ChIP-exo) and RNA sequencing (RNA-seq). From this analysis, we identified and validated sequences of the SetCD operators. Finally, we investigated the dynamics of integration and excision of SXT to address the regulation of expression of *xis* and *int* in both donor and recipient cells. We demonstrated that SetCD must be expressed *de novo* in the recipient cells to allow the establishment of SXT in a new host.

## MATERIALS AND METHODS

### Bacterial strains and bacterial conjugation assays

The *E. coli* strains used in this study, all derivatives of CAG18439 or BW25113 (29,30), are described in Table 1. The strains were routinely grown in Luria-Bertani (LB) broth at 37°C in an orbital shaker/incubator and were maintained at −80°C in LB broth containing 15% (vol/vol) glycerol. Antibiotics were used as described in Text S1. Conjugation assays were performed as described elsewhere (31). To induce expression of Int from pInt33 and SetCD from pGG2B in complementation assays, mating experiments were carried on LB agar plates supplemented with 0.02% arabinose (32).

**Table 1.** Strains and plasmids used in this study

Strain or plasmid	Relevant genotype and phenotype^a^	Reference
*E. coli* strains		
CAG18439	MG1655 *lacZU118 lacI42*::Tn*10* (Tc^r^)	(29)
BW25113	F-, Δ(*araD*-*araB*)567, Δ*lacZ4787*(::*rrnB-3*), λ-, *rph-1*, Δ(*rhaD*-*rhaB*)*568, hsdR514*	(30,33)
HW220	CAG18439 *prfC*::SXT (Tc^r^ Su^r^ Tm^r^)	(24)
VB17	BW25113 *prfC*::SXT (Su^r^ Tm^r^)	This study
AD57	CAG18439 *prfC*::ICE*Vfl*Ind1 *yicC::*DUP(MGI*Vfl*Ind1) (Tc^r^ Su^r^ Tm^r^)	(22)
AD72	CAG18439 *prfC*::SXT *yicC*::MGI*Vfl*Ind1*::aph* (Tc^r^ Su^r^ Tm^r^ Kn^r^)	(22)
AD133	CAG18439 *prfC*::[SXT Δ*setCD*] *yicC*::MGI*Vfl*Ind1::*aph* (Tc^r^ Su^r^ Tm^r^ Kn^r^)	(23)
AD132	CAG18439 *yicC*::MGI*Vfl*Ind1::*aph* pGG2B (Tc^r^ Kn^r^ Ap^r^)	(23)
DPL2	CAG18439 *prfC*::SXT pGG2B (Tc^r^ Su^r^ Tm^r^ Ap^r^)	This study
DPL3	CAG18439 *prfC*::[SXT Δ*setCD*] (Tc^r^ Su^r^ Tm^r^)	This study
DPL5	CAG18439 *prfC*::[SXT Δ*setCD*] pGG2B (Tc^r^ Su^r^ Tm^r^ Ap^r^)	This study
VB111	MG1655 (Nx^r^)	(34)
DPL9	VB111 pInt33 (Nx^r^ Ap^r^)	This study
DPL246	BW25113 *prfC*::[pDPL227 Δ*setR*::*lacZ*-*aad7*] (Cm^r^ Sp^r^)	This study
DPL453	BW25113 *attB*_λ_::pDPL440 pGG2B (Kn^r^ Ap^r^)	This study
DPL400	BW25113 *attB*_λ_::pDPL382 pGG2B (Kn^r^ Ap^r^)	This study
DPL490	BW25113 *attB*_λ_::pDPL465 pGG2B (Kn^r^ Ap^r^)	This study
DPL393	BW25113 *attB*_λ_::pDPL384 pGG2B (Kn^r^ Ap^r^)	This study
DPL394	BW25113 *attB*_λ_::pDPL385 pGG2B (Kn^r^ Ap^r^)	This study
DPL489	BW25113 *attB*_λ_::pDPL467 pGG2B (Kn^r^ Ap^r^)	This study
DPL494	MG1655 *lacZo*^CD1^	This study
DPL501	MG1655 *lacZo*^CD2^	This study
DPL513	BW25113 *prfC*::R391 (Kn^r^)	This study
DPL491	BW25113 *prfC*::[R391 *orf90*^3xFLAG^] (Kn^r^)	This study
DPL492	BW25113 *prfC*::[SXT *setC*^3xFLAG^] (Su^r^ Tm^r^)	This study
DPL493	CAG18439 *prfC*::[ICE*Vfl*Ind1 *setC*^3xFLAG^] *yicC*::DUP(MGI*Vfl*Ind1) (Tc^r^ Su^r^ Tm^r^)	This study
Plasmids		
pInt33	pBAD33 *int*_SXT_ (Cm^r^)	(25)
pBAD30	*ori* p15A *bla araC P*_BAD_-MCS (Ap^r^)	(32)
pGG2B	pBAD30 *setCD* (Ap^r^)	(39)
		
pDPL189	pCR2.1-TOPO *attP*_SXT_ (Ap^r^ Kn^r^)	This study
pKD3	PCR template for one-step chromosomal gene inactivation (Cm^r^)	(33)
pKD13	PCR template for one-step chromosomal gene inactivation (Kn^r^)	(33)
pVI36	PCR template for one-step chromosomal gene inactivation (Sp^r^)	(34)
pDPL458	pKD3 containing −73 to −31 of *P_s003_* (Cm^r^)	This study
pNC12	pVI36-SPATagCt (Sp^r^)	This study
pOPlacZ	*ori*_R6Kγ_*attP*_λ_*aph lacZ* (Kn^r^)	(35)
pDPL440	pOPlacZ containing −100 to +81 of SXT *P_s003_* (Kn^r^)	This study
pDPL382	pOPlacZ containing −52 to +81 of SXT *P_s003_* (Kn^r^)	This study
pDPL465	pOPlacZ containing −35 to +81 of SXT *P_s003_* (Kn^r^)	This study
pDPL384	pOPlacZ containing −180 to +11 of SXT *P_xis_* (Kn^r^)	This study
pDPL385	pOPlacZ containing −52 to +11 of SXT *P_xis_* (Kn^r^)	This study
pDPL467	pOPlacZ containing −35 to +11 of SXT *P_xis_* (Kn^r^)	This study

^a^Ap^r^, ampicillin resistant; Cm^r^, chloramphenicol resistant; Em^r^, erythromycin resistant; Kn^r^, kanamycin resistant; Nx^r^, nalidixic acid resistant; Sp^r^, spectinomycin resistant; Su^r^, sulfamethoxazole resistant; Tc^r^, tetracycline resistant; Tm^r^, trimethoprim resistant; Ts, thermosensitive.

### Molecular biology methods

Genomic and plasmid DNA preparation, PCR products amplification and purification, electro-transformation of *E. coli*, gene expression analysis by quantitative real-time PCR (qRT-PCR) and β-galactosidase assays, Southern blotting, contour-clamped homogeneous electric field pulsed field gel electrophoresis (CHEF-PFGE) and sequencing were performed using standard molecular biology techniques. Details are provided in Text S1.

### Plasmid and strain constructions

Plasmids and primers used in this study are described in Table 1 and Supplementary Table S2, respectively. Mutants of SXT, R391 and ICE*Vfl*Ind1 were constructed using the one-step chromosomal gene inactivation technique using pKD3, pKD13 and pVI36 as templates (33,34). Constructions of reporter and expression vectors were done using conventional molecular methods. Detailed methodology is described in Text S1.

### ChIP-exo experiments and RNA sequencing

The ChIP-exo, RNA-seq and genome-wide 5′ rapid amplification of cDNA ends (5′ RACE) experiments were conducted as described in Carraro *et al*. (35). Additional details are provided in Text S1. Sequenced libraries are described in Supplementary Table S3.

### Data availability

Fastq files for each experiment were deposited at the NCBI Sequence Read Archive under accession numbers SRX708080 and SRR1583172 for SXT ChIP-exo, SRX708425 and SRR1583516 for R391 ChIP-exo, SRX708426 and SRR1583532 for ICE*Vfl*Ind1/MGI*Vfl*Ind1 ChIP-exo, SRX708086 and SRR1583199 for SXT RNA-seq as well as SRX708115 and SRR1583202 for SXT 5′-RACE. Complete data from aligned reads for ChIP-exo, RNA-seq and 5′-RACE can also be visualized using the UCSC genome browser at http://bioinfo.ccs.usherbrooke.ca/setCD.html.

## RESULTS

### Characterization of the SetCD regulon in three SXT/R391 ICEs

The exact target genes and sequence motif recognized by the SetCD complex, which plays an essential role in ICE conjugative transfer activation, has yet to be determined. Using RNA-seq and ChIP-exo (35,36), we undertook an exhaustive characterization of the SetCD regulon in three ICEs found in clinical isolates of three different pathogens: the prototypical ICEs SXT from *V. cholerae* O139 (19) and R391 from *Providencia rettgeri* (37) as well as ICE*Vfl*Ind1 from *Vibrio fluvialis* (38). *E. coli* strains DPL492, DPL491 and DPL493 (Table 1) bearing derivatives of SXT, R391 and ICE*Vfl*Ind1, each expressing a native SetD subunit along with a SetC subunit C-terminally fused to the 3xFLAG tag (SetC^3xFLAG^), were used in these experiments (Supplementary Figure S1A). The 3xFLAG tag did not affect the function of the SetC orthologs based on the similar transfer frequency of each ICE relative to its wild-type counterpart (Supplementary Figure S1B).

The ChIP-exo and RNA-seq assays were carried out after induction of the cell cultures using mitomycin C to trigger expression of SetCD from its native promoter. ChIP-exo data analyses revealed nine major SetCD enrichment peaks located upstream of the same genes and operons—most of which playing a key role in conjugative transfer—in the conserved core sequence shared by the three ICEs (Figure 1A, B and Supplementary Table S1). Four of these peaks are located upstream of genes that are predicted to be involved in the formation of the mating pore: *traL* (conjugal transfer pilus assembly protein, peak V), *traV* (outer membrane lipoprotein, peak VI), *dsbC* (conjugative disulfide bond isomerase, peak VII) and *traF* (conjugal pilus assembly protein, peak IX). One peak was also present inside the 3′ end of the predicted relaxase gene *traI*, upstream of the gene *traD* (type IV coupling protein, peak IV). Two additional peaks were observed, one upstream of *xis* (peak I) and one (peak II) in the intergenic region between *mobI* (auxiliary component of the relaxosome) and *s003*, which is part of the operon containing *int*. The strongest peak (peak VIII) was detected in the intergenic region between the two divergent genes *s063* and *s089*. *s089* is the first gene of a large operon coding for a RecA-independent homologous recombination system (39,40). The last statistically significant peak (peak III) is located in the intergenic region between *rumA* (UmuD-like protein) and *s024*. Transcriptional activity measured by RNA-seq in *E. coli* HW220 (wild-type SXT) and DPL3 (SXT Δ*setCD*) correlates with the presence of a SetCD-binding site as the expression of 29 out of 52 core genes in SXT, including genes located downstream of SetCD-binding sites, is significantly increased upon mitomycin C induction compared to a Δ*setCD* mutant under the same conditions (Figure 1C and Dataset S1). Most genes that are not significantly affected by the expression of SetCD in SXT appear to be either inactive or constitutively expressed, and are mainly found in variable cargo DNA. Their functions are unknown or not directly tied to conjugative transfer, and include the antibiotic resistance genes, transposase genes, the *s027*-*s040* gene cluster, the diguanylate cyclase gene *dgcL* among others (Dataset S1).

### Characterization of SetCD-dependent promoters

**Figure 2.**
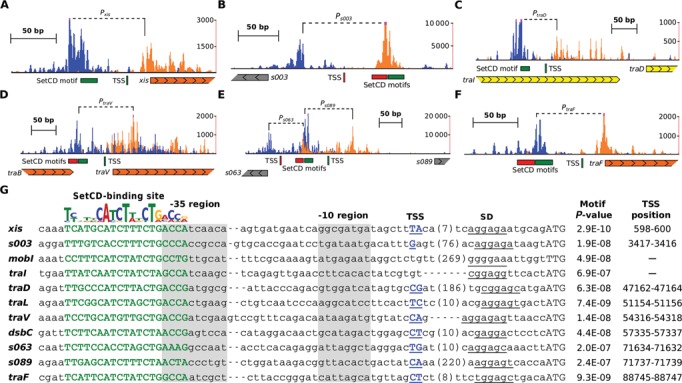
Organization of SetCD-dependent promoters. (**A–F**) SetCD-binding profile by ChIP-exo represented by the number of reads mapped on the promoter region upstream of *xis* (A), *s003* (B), *traD* (C), *traV* (D), *traF* (F) and in the intergenic region between *s063* and *s089* (E). The tracks plot ChIP-exo reads density (positive DNA strand in blue, negative DNA strand in orange) at single nucleotide resolution. SetCD-binding motifs and TSS are indicated (positive DNA strand in green, negative DNA strand in red). The TSS of *s089* was previously identified (39). Genes are represented by arrows and are color coded based on their function as in Figure 1. (**G**) Alignment of SetCD-dependent promoters in SXT. The SetCD-binding logo of SXT is reproduced from Figure 1D. SetCD boxes are shown in bold green capital letters with their respective *P*-value. The position of the TSS in the promoter sequences is indicated when available (bold blue capital letters and underlined) along with the corresponding coordinates in SXT. Shine–Dalgarno sequences (SD) are underlined while start codons are in capital letters. The approximate position of the −35 and −10 regions is highlighted in gray. Numbers in bracket indicate the length in bp of spacers between the represented TSS and the SD sequence.

We carried out *de novo* motif discovery of DNA sequences bound by SetCD for each independent ICE ChIP-exo dataset, thereby generating three highly similar logo sequences (Figure 1D) in which subtle differences between the extracted motifs reflect ICE-specific polymorphisms in promoter regions. For each ICE, we next determined the exact location of proposed SetCD-binding sites within the ChIP-exo peaks (Figure 1B) and observed a footprint likely corresponding to the SetCD and RNA polymerase holoenzyme complexes bound to the corresponding promoters (Figure 2A–F, and Supplementary Table S1) (35,41). In some instances, we were able to identify two occurrences of a SetCD motif within the same peak. For example, the intergenic region *s063*–*s089* contains back-to-back SetCD-binding motifs, thereby revealing the presence of two SetCD-activated divergent promoters (Figure 2E). 5′-RACE and primer extension analyses (Figure 1C and Supplementary Figure S2) allowed us to determine transcription start sites (TSS) in SXT, revealing that SetCD-binding motifs are located immediately upstream of the −35 promoter box. This promoter structure was observed for all TSS located between a SetCD-binding motif and a gene in the same orientation (Figure 2G). This organization is reminiscent of class II activation, in which the activator binds to a sequence that overlaps the promoter −35 element and usually contacts the RNA polymerase σ subunit (41).

### Validation of the SetCD operator sequences

**Figure 3.**
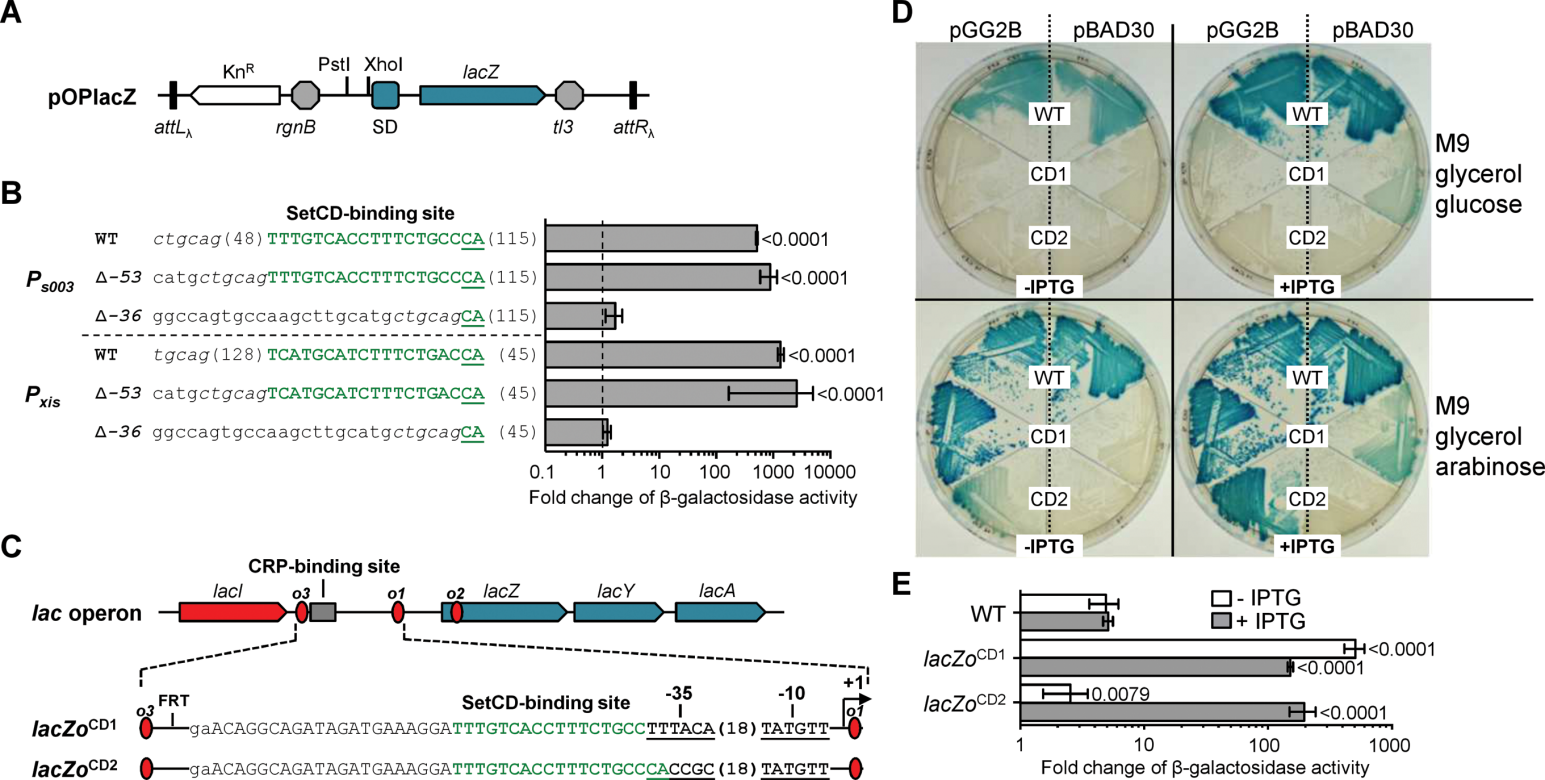
Validation of the DNA motif mediating the activation by SetCD. (**A**) Schematic representation of pOPlacZ, an integrative vector used as a promoter activity reporter system. Promoter regions were cloned between the PstI and XhoI restriction sites upstream of a promoterless *lacZ* gene transcriptionally isolated by the terminator sequences *rgnB* and *tl3*. SD, Shine-Dalgarno sequence. (**B**) β-galactosidase activity measured for *P_s003_* and *P_xis_* (WT) and derivatives Δ*-53* and Δ*-36* with SetCD expressed from an arabinose-inducible promoter on pGG2B. Nucleotides from the SetCD boxes are shown in green. Promoter variants were transcriptionally fused to *lacZ* in the pOPlacZ vector and integrated in one copy at the *attB*_λ_ site of *E. coli* BW25113 (Table 1). The right panel reports β-galactosidase activities expressed as the ratio between the Miller units in the arabinose-induced versus non-induced conditions. Results are the means and standard deviations of at least three independent biological replicates. The *P*-values from a two-way ANOVA with Tukey's multiple comparison test comparing the log of the means of the *P_s003_* and *P_xis_* WT or Δ*-53* variants relative to the corresponding Δ*-36* mutant are indicated. (**C**) Organization of two SetCD-dependent mutants of *P_lac_*. In both mutants the CRP-binding site was replaced by the SetCD operator of *P_s003_*. *lacZo*^CD1^ carries the −35 of *P_lac_*, while *lacZo*^CD2^ has the −35 of *P_s003_*. (**D**) Wild-type *E. coli* MG1655 (WT), *lacZo*^CD1^ mutant DPL494 (CD1) and *lacZo*^CD2^ mutant DPL501 (CD2) grown on M9 glycerol minimal medium supplemented with glucose or arabinose, with or without IPTG. The strains were carrying either pBAD30 or its *setCD*-expressing derivative pGG2B. (**E**) β-galactosidase activity measured for *E. coli* MG1655, DPL494 (*lacZo*^CD1^ mutant) and DPL501 (*lacZo*^CD2^ mutant) containing pGG2B grown in M9 glycerol minimal medium with glucose or arabinose, with or without IPTG. Ratios between the Miller units in the arabinose-induced versus glucose conditions are shown. Results are the means and standard deviations of four independent biological replicates. The *P*-values from a two-way ANOVA with Tukey's multiple comparison test comparing the log of the means of the *lacZo*^CD1^ and *lacZo*^CD2^ mutants relative to the wild-type in the corresponding conditions are indicated.

We validated that the proposed SetCD-binding motifs promote the observed binding of SetCD and transcriptional activation by fusing the *lacZ* reporter gene to the promoters *P_s003_* (DPL453) and *P_xis_* (DPL393) that are responsible for the expression of *int* and *xis*, respectively. For each promoter, two mutants were also generated. A first variant, Δ*-53*, lacked the sequence immediately upstream of the promoter-proximal SetCD motif (DPL400 and DPL394) while another variant, Δ*-36*, lacked the entire region upstream of the −35 promoter box, thus removing the first 17 bp of the proximal SetCD box (DPL490 and DPL489) (Figure 3A and B). β-galactosidase assays were then carried out upon *setCD* expression from the arabinose-inducible *P*_BAD_ promoter. Addition of the predicted SetCD boxes of *P_s003_* and *P_xis_* upstream of *lacZ* boosted the β-galactosidase activity by respectively ∼520- and 2 200-fold, while the presence of additional upstream sequence made no difference (Figure 3B, compare WT with Δ*-53*). When the putative SetCD boxes were missing, the β-galactosidase activity dropped below the detection limit of our assay (Figure 3B, compare Δ*-53* with Δ*-36*).

To confirm that this motif alone, and not a hypothetical factor acting in *cis*, was sufficient to confer SetCD-dependent induction of gene expression, we substituted the native-binding site of the cAMP receptor protein (CRP) of the *P_lac_* promoter upstream of *lacZYA* in *E. coli* MG1655 by the SetCD box of *P_s003_*. Two chimeric promoters containing the operator mutations *lacZo*^CD1^ and *lacZo*^CD2^ were constructed (Figure 3C). The *lacZo*^CD1^ mutant (DPL494) retained the −35 element of *P_lac_*, whereas it was substituted by the −35 element of *P_s003_* in the *lacZo*^CD2^ mutant (DPL501). The three operator sites *o*_1_, *o*_2_ and *o*_3_ of the LacI repressor remained unaffected in both constructs. *setCD* was expressed under the control of *P*_BAD_ from pGG2B in the strains containing the constructions and in wild-type MG1655. The absence of significant β-galactosidase activity observed on M9 glycerol medium supplemented with glucose for both *lacZo*^CD1^ or *lacZo*^CD2^ mutations confirmed the inability of the hybrid promoters to respond to the activation by CRP bound to cAMP regardless of the alleviation of LacI repression by IPTG and of the presence of repressed *setCD* (Figure 3D). In contrast, when glucose was replaced by arabinose, expression of *setCD* triggered a strong expression from the promoter containing *lacZo*^CD1^, producing dark blue colonies, and weak expression from the one containing *lacZo*^CD2^ (Figure 3D and E). Strong expression from the latter was observed only upon concomitant alleviation of LacI repression by addition of IPTG (Figure 3D and E). These results indicate that expression of the *lacZYA* operon became SetCD-dependent when the CRP operator site of *P_lac_* was replaced by either *lacZo*^CD1^ or *lacZo*^CD2^. Interestingly, the variant *lacZo*^CD2^ seemed to remain strongly repressed by LacI upon *setCD* overexpression as shown by the lack of induction in the absence of IPTG, while the variant *lacZo*^CD1^ was not (Figure 3E). This phenotypical difference observed between the two mutants can be attributed to their respective −35 sequence. The −35 of *lacZo*^CD2^ (CACCGC) is very distant from the σ^70^ consensus, while *lacZo*^CD1^ harbors the more canonical −35 of *P*_lac_ (TTTACA). Taken together, these experiments confirm that the ChIP-exo derived SetCD motif alone is sufficient to confer SetCD-dependent activation of gene expression.

### ChIP-exo assays reveal SetCD-regulated conserved genes in MGIs

**Figure 4.**
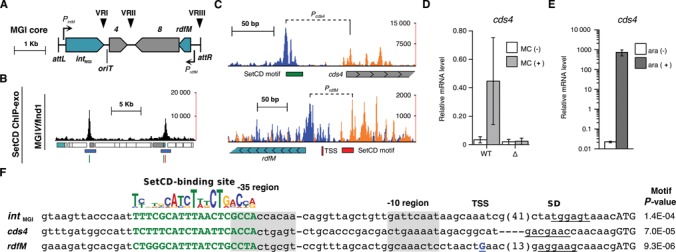
SetCD-dependent activation of MGI*Vfl*Ind1. (**A**) Representation of the conserved genes of MGIs. The color code is the same as in Figure 1A. (**B**) SetCD ChIP-exo binding profile in MGI*Vfl*Ind1. Tracks are organized and color coded as in Figure 1B. SetCD motifs were derived from the SetCD-binding logo of ICE*Vfl*Ind1 presented in Figure 1D. (**C**) Close-up view of the peaks located in the intergenic region upstream of *cds4* and *rdfM* in MGI*Vfl*Ind1. Tracks are organized as in Figure 2A–F. Genes are represented by arrows and are color coded as in Figure 1B. (**D**) Quantification by qRT-PCR of the mRNA transcripts of *cds4* in *E. coli* strains containing wild-type SXT (WT; AD72) or SXT Δ*setCD* (Δ; AD133) in the presence or absence of mitomycin C (MC). (**E**) The effect of arabinose-induced SetCD expression from pGG2B on *cds4* mRNA in an *E. coli* strain devoid of ICE (AD132) as determined by qRT-PCR. (**F**) Alignment of SetCD-dependent promoters in MGI*Vfl*Ind1. The SetCD logo of ICE*Vfl*Ind1 is represented as in Figure 1D. Sequences are organized as in Figure 2G. The *int*_MGI_ SetCD-binding motif was found by FIMO while *cds4* and *rdfM* motifs were found by MAST. The TSS of *rdfM* was determined by primer extension (Supplementary Figure S2) and is located at position 18 284–18 285 on the negative DNA strand.

The strain DPL493 used for ChIP-exo assays also contains MGI*Vfl*Ind1, an MGI originally detected in the same *V. fluvialis* strain that contains ICE*Vfl*Ind1 (Table 1), allowing us to monitor on this MGI the binding of SetCD provided in *trans* by ICE*Vfl*Ind1. Two major peaks were detected on MGI*Vfl*Ind1, both mapping upstream of two of the four conserved core genes (Figure 4A–C and Supplementary Table S1). The first one was detected upstream of *rdfM* and the second one was found upstream of *cds4*, a gene of unknown function. To test whether *cds4* is regulated by SetCD, we measured its expression by qRT-PCR in *E. coli* containing SXT (AD72), SXT Δ*setCD* (AD133) or pGG2B, the plasmid expressing SetCD from *P*_BAD_ (AD132). While expression of *cds4* was induced by mitomycin C in the strain containing wild-type SXT, it was nearly abolished in the absence of *setCD* regardless of the presence of mitomycin C (Figure 4D). Overexpression of SetCD alone also dramatically increased the level of *cds4* transcript (∼30 000-fold induction, Figure 4E), thereby confirming that *cds4* of MGIs is activated by SetCD. Motif Alignment and Search Tool (MAST) analysis on MGI*Vfl*Ind1 sequence revealed the presence of SetCD-binding motifs upstream of *cds4* and *rdfM* (Figure 4B, C and F). Mapping of the TSS of *rdfM* obtained by primer extension analysis (Supplementary Figure S2) suggests that like in SXT, SetCD acts as a class II activator in MGIs (Figure 4C and F).

### Establishment of SXT into a naive host requires *de novo* expression of *setCD*

Although it is known, and confirmed by our results, that SetCD activates the expression of SXT *int* in the donor strain (20,25), the importance of SetCD for the expression of *int* in recipient cells and consecutive integration of SXT in the recipient's chromosome is not clearly established as conflicting observations have been reported. Results from Beaber *et al*. (20) suggest that *setCD* is expendable in the recipient cells as Δ*setC* and Δ*setD* mutants of SXT have been shown to transfer and establish in recipient cells when the deletions were complemented in the donor cells exclusively. If SetC and SetD are not necessary for integration of SXT in the chromosome of the recipient cells, then expression of *int* likely occurs at a low constitutive level. Alternatively, SetCD could be produced in the donor cell and translocated through the mating pore into the recipient cell during conjugative transfer to stimulate *int* expression in the recipient. However, Burrus and Waldor reported that a suicide vector containing *attP* and *int* driven by its native promoter (*P_s003_*) is unable to integrate in recipient cells lacking *setCD* (25), thereby suggesting that SetCD is required for *de novo* expression of *int* in recipient cells.

**Figure 5.**
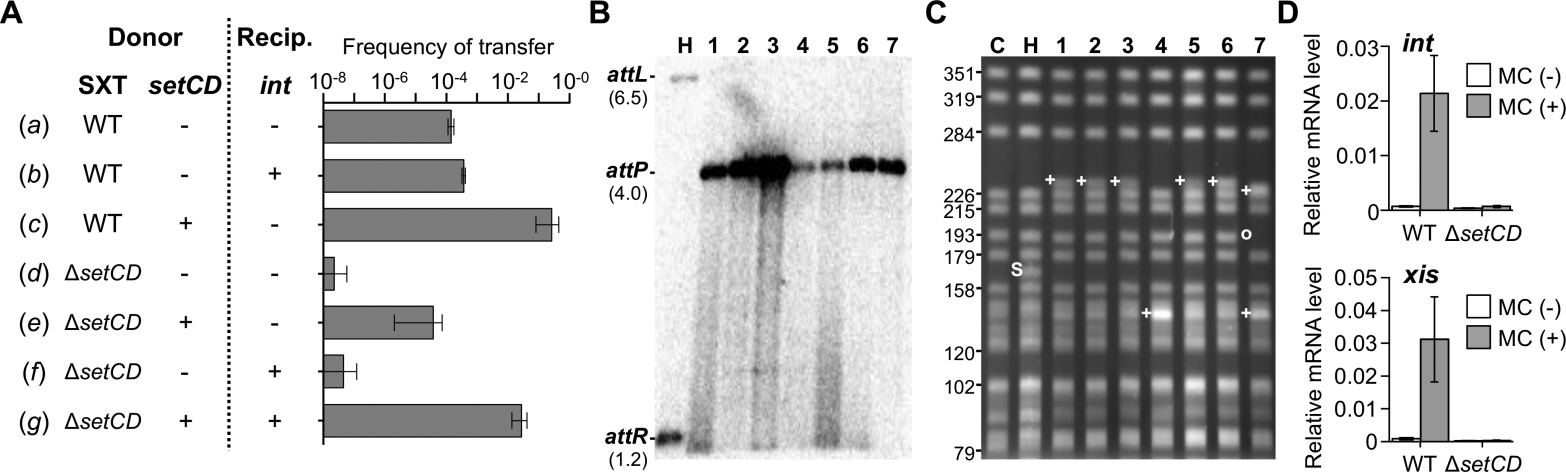
*De novo setCD* expression is required for normal establishment of SXT in a new host. (**A**) Conjugation assays using as donor *E. coli* CAG18439 derivatives harboring SXT (WT, HW220 or DPL2) or its Δ*setCD* mutant without (-) or with (+) *setCD* expressed from pGG2B (DPL3 or DPL5). The recipient strain is an Nx^r^-derivative of *E. coli* MG1655 expressing (+) or not (-) *int* under control of *P*_BAD_ from pInt33 (DPL9 or VB111). Results are the mean values and standard deviations of three independent biological replicates. An unpaired t-test with Welch's correction was used to compare the log of the mean values. *P*-values are as follow: *a* versus *c*, <0.0001; *a* versus *d*, 0.0916; *a* versus *g*, <0.0001; *c* versus *g*, 0.0064. (**B**) Southern blot hybridization analysis of EcoRI/EcoRV-digested genomic DNAs of seven randomly picked exconjugants isolated from mating *e* (DPL5 × VB111) in panel A probed with the *attP* fragment of SXT (lanes 1 to 7). Lane H, donor strain HW220. (**C**) Ethidium bromide-stained agarose gel of SpeI-digested genomic DNAs separated by contour-clamped homogeneous electric field pulsed field gel electrophoresis (CHEF-PFGE). Molecular sizes are in kilobases. (S), fragment containing SXT inserted in *prfC*; (+) and (o), respectively, atypical and missing fragments compared to control lanes C and H. Lanes: C, CAG18439; H, HW220; 1 to 7, randomly picked exconjugants isolated from mating *e* in panel A. (**D**) Quantification by qRT-PCR of the mRNA transcripts containing *int* and *xis* in CAG18439 containing wild-type SXT (WT; HW220) or SXT Δ*setCD* (DPL3) in the presence or absence of mitomycin C (MC).

To clearly address the role of SetCD in the establishment of SXT in the recipient cells, we conducted mating assays using combinations of *E. coli* donor and recipient strains harboring SXT or its Δ*setCD* mutant with or without plasmids expressing either *int* or *setCD* under control of *P*_BAD_ (Figure 5A). Overexpression of *int* in recipient cells did not enhance the transfer of SXT (Figure 5A *a* and *b*), indicating that SXT integration into the recipient's chromosome is not a rate-limiting step. This conclusion is also supported by *setCD* overexpression in the donor cell, which resulted in a ∼3-log increase of transfer (Figure 5A *a* and *c*). Consequently, mating pore assembly or DNA translocation is likely the rate-limiting step of SXT conjugative transfer. As expected, transfer of the Δ*setCD* mutant was abolished, even upon expression of *int* in recipient cells (Figure 5A *d* and *f*).

Intriguingly, transfer of SXT Δ*setCD* was only partially restored when *setCD* was overexpressed in donor cells, at a rate of only one-fifth of wild-type SXT and ∼4 logs lower than wild-type SXT upon *setCD* overexpression (Figure 5A, compare *a* versus *e* and *c* versus *e*). Because in such a context, expression of the transfer genes is not compromised in donor cells, the low rate of transfer of mating *e* suggests that integration of SXT Δ*setCD* into the recipient's chromosome became the rate-limiting step of transfer. Assuming that this phenotype was attributable to weak or lack of expression of *int* in the recipient cells, we can rule out that the SetCD protein complex is translocated, at least in significant amounts, from the donor to recipient cells during SXT transfer. Indeed, stimulation of *int* expression mediated by translocated SetCD should have enabled normal integration of SXT Δ*setCD* at a wild-type rate despite the lack of *setCD* genes in the recipient cells. In fact, transfer of SXT Δ*setCD* was fully restored to wild-type level only upon concomitant overexpression of *setCD* in the donor and *int* in the recipient (Figure 5A *g* and *c* versus *e*). Altogether, these results suggest that the SetCD complex is not translocated from the donor to recipient cells during conjugation. Instead, *setCD* is expressed *de novo* upon entry of SXT in the recipient cells, allowing *int* expression to mediate SXT integration.

### A *setCD*-null mutant of SXT maintains atypically in exconjugant colonies

The Δ*setCD* mutation seemed to hinder the expression of *int* in the recipient cells, eventually leading to loss of the incoming SXT. Suboptimal *int* expression could reduce integration or promote maintenance of the ICE by other means. We submitted a sample of seven Δ*setCD* exconjugants randomly picked from mating *e* (Figure 5A) to profiling by Southern blot hybridization and PFGE analyses. Southern blot probing of the genomic DNA of these exconjugants with a fragment overlapping *attP* revealed atypical restriction patterns compared to the control donor strain containing SXT integrated as a single copy into *prfC* (HW220) (Figure 5B). All exhibited a signal for *attP* but lacked the characteristic *attL* and *attR* fragments normally present after correct SXT integration, suggesting that SXT Δ*setCD* failed to integrate site-specifically. This result was also supported by PCR amplification of *attB*, which confirmed that the 5′ end of *prfC* was intact in all seven exconjugants (Supplementary Figure S3A and B). At least two possible mechanisms could explain the formation of such anomalous exconjugants. First, SXT Δ*setCD* could have integrated by homologous recombination or transposition potentially through one of the insertion sequence present in the variable region VRIII (Figure 1A). Alternatively, SXT Δ*setCD* could maintain as a circular replicative molecule. We tested both hypotheses by subjecting the genomic DNA of the same exconjugants to SpeI restriction, which does not cut SXT, and PFGE. None of the exconjugants exhibited the expected 166-kb SpeI fragment containing SXT as seen in the control donor strain HW220 (Figure 5C). Instead distinct restriction patterns were observed. In the most frequent pattern (five out of seven exconjugants), a new large fragment of ca. 230 kb was observed. This is inconsistent with the possibility of tandem integration of multiple copies of SXT at *prfC* since two copies of SXT would result in a larger 257-kb SpeI fragment (31) suggesting that SXT Δ*setCD* integrated into different chromosomal loci. None of these patterns exhibited a 99-kb band compatible with a replicative form of SXT. Altogether these results confirmed that SXT Δ*setCD* is unable to integrate into *prfC* in a site-specific fashion upon entry into a naive host, supporting that *int* expression was compromised or abolished in Δ*setCD* mutants.

### Expression of *int* and *xis* requires activation by SetCD

The defective integration phenotype of SXT Δ*setCD* suggests that expression of *int* strictly depends upon activation by SetCD. To test this hypothesis, we monitored the expression of *int* by qRT-PCR on cDNA derived from *E. coli* strains containing SXT (HW220) or its Δ*setCD* derivative (DPL3). We also monitored the expression of *xis* in the same conditions as it is expected to be expressed only upon activation of SXT transfer to promote the site-specific excision reaction over integration. The DNA-damaging agent mitomycin C increased *int* and *xis* mRNA transcript levels by 31- and 39-fold, respectively, in a *setCD*-dependent fashion (Figure 5D), thereby supporting our RNA-seq results showing that their expression is controlled by SetCD. Furthermore, both *int* and *xis* transcript levels were slightly above the limit of detection for wild-type SXT in non-inducing conditions (Figure 5D). Spontaneous induction of the SOS response in a subpopulation of the cell culture (42) likely accounts for this low basal level of expression and for the basal level of SXT transfer (∼5×10^−4^ exconjugant/recipient in Figure 5A *a*). In the same conditions, expression of both *int* and *xis* dropped below the detection level for SXT Δ*setCD* regardless of the presence or absence of mitomycin C (Figure 5D). Together with the failure of the *setCD*-null mutant of SXT to integrate into *prfC* of recipient cells, these results demonstrate that *de novo* expression of *setCD* is required to trigger the expression of *int* in recipient cells and allow stable maintenance and inheritance of SXT/R391 ICEs.

## DISCUSSION

**Figure 6.**
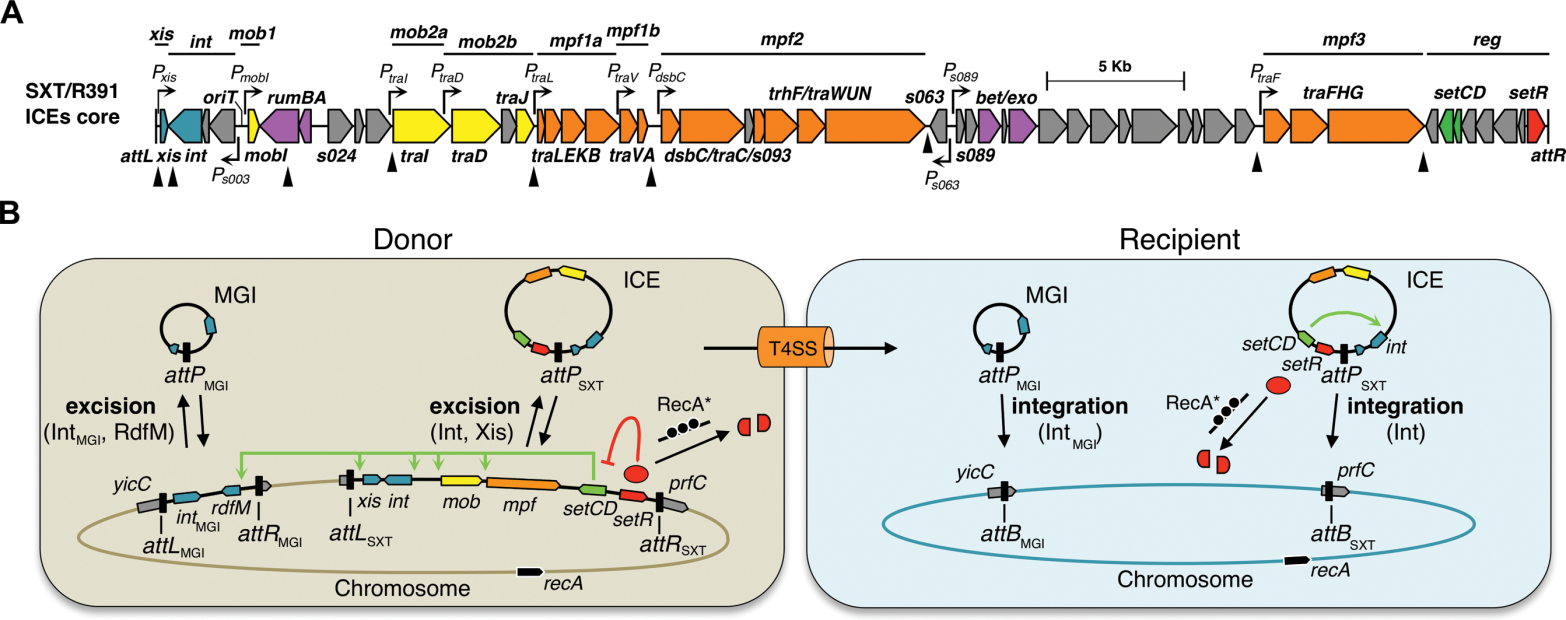
(**A**) Overview of the SetCD-regulated promoters on the conserved core of genes of SXT/R391 ICEs. Genes and annotations are color coded as in Figure 1A, and black triangles show the position of variable cargo DNA. (**B**) Summary of the main regulatory events occurring in the donor and recipient cells during conjugative transfer of SXT/R391 ICEs and MGIs.

In many archetypical conjugative elements such as the IncFI F conjugative plasmid or the ICE Tn*916*, genes coding for the conjugative apparatus are organized as a single polycistronic operon (43,44). In contrast, although the *tra* genes of SXT/R391 ICEs are syntenic with the *tra* genes of F-like conjugative plasmids (20,43), they are distributed in five distinct gene clusters, separated by variable DNA clusters (Figure 1A) (6). Such an organization requires an adaptable activation system that allows coherent expression of the diverse components essential for ICE conjugation. This includes activation of the site-specific recombination system mediating excision from the chromosome as a circular molecule (*xis* and *int*), assembly of the mating apparatus (*traLEKBVA, dsbC*/*traC*/*trhF*/*traWUN* and *traFHG*), initiation of ICE DNA transfer (*mobI*/*oriT* and *traIDJ*) and finally integration into the recipient cell's chromosome (*int*) (Figure 6A).

In the present study we have established using ChIP-exo and RNA-seq experiments that SetCD coordinate the expression of many genes and operons by binding upstream of the TSS of *xis, s003, mobI, traI, traD, traL, traV, dsbC, s063, s089* and *traF* (Figure 6A). This transcriptional regulation profile is similar to the one recently reported for the conjugative transfer functions encoded by the IncA/C conjugative plasmids that are activated by AcaCD, an activator complex distantly related to SetCD (34% and 23% identity between the C and D subunits, respectively) (35). In fact, activation of gene expression by SetCD and AcaCD seems to be reminiscent of the activation of transcription by the transcriptional master regulator of bacterial flagellum biogenesis FlhCD. The complex FlhC_2_D_2_ binds 30–bp upstream of the σ^70^-dependent TSS and activates the expression of class II ﬂagellar operons, which encode components of the ﬂagella basal body and export machinery (45). O'Halloran *et al*. (46) previously reported a predicted potential SetCD-binding site upstream of *xis* in R391 based on analogies with ‘FlhD_2_C_2_ box’ arms. However, no such a binding motif exists upstream of *xis* in ICEs of the SXT/R391 family as the SetCD DNA recognition motif obtained by combining the SetCD-bound promoters of SXT, R391 and ICE*Vfl*Ind1 drastically differs from the FlhD_2_C_2_ (47–50) and AcaCD (35) binding sites. This is not surprising given the low similarity between SetCD, FlhCD and AcaCD (20,35). The −35 and −10 elements of all SetCD-dependent promoters studied here are poorly conserved relatively to the σ^70^ canonical −35 and −10 promoter boxes (51,52). In fact, the −35 region lacks a recognizable motif of the canonical −35 signal (TTGACA). In all the SetCD-dependent promoters, we found that the SetCD box overlaps or is located immediately upstream of the −35 element (Figures 2G and 4F). The close proximity of the SetCD box near the sequence usually recognized by σ factors suggests that binding of SetCD compensates for the lack of a recognizable −35 element, allowing recruitment of the RNA polymerase holoenzyme, in a manner similar to the class II CRP-, FNR- and FlhD_2_C_2_-dependent promoters (53–55). Biochemical characterization of SetCD is needed to clarify whether SetCD operates like FlhD_2_C_2_ by interacting with the RNA polymerase α subunit C-terminal domain (56).

Our data allowed us to deepen our understanding of the regulation of ICEs of the SXT/R391 family. To date both *traIDJ* and *traLEKBVA* were presumed to be two single polycistronic operons each regulated by a unique promoter located upstream of *traI* and *traL*, respectively (6,20). To our surprise, our data indicate that expression of these two gene clusters is much more complex. First, the promoter of *traI* differs between ICEs because the −10 element is part of the conserved core whereas the −35 region is brought by variable DNA found in the variable region HS5 (Figure 1A and Supplementary Figure S4B). Yet clearly, *traI* expression is dependent of SetCD in SXT as shown by our RNA-seq data (Dataset S1) and close examination of the ChIP-exo signal in the region upstream of *traI* suggests the presence of a potential weaker promoter footprint as well as a degenerate SetCD-binding motif for all three ICEs (Supplementary Figure S4A and Table S1). This raises interesting questions about the selective pressure operating on sequences inserting near the −35 promoter element of *traI* for the conservation of a functional SetCD operator. Second, *traD* expression is driven from a SetCD-dependent promoter located within *traI* (Figure 2C and G). Finally, the *traLEKBVA* gene cluster likely corresponds to two independent operons, *traLEKB* and *traVA*, although we cannot rule out the existence of mRNA transcripts containing *traL* to *traA* (Figures 1A and C, 2D and G). ChIP-exo data revealed a strong peak and a well-conserved SetCD motif within the 5′ end of the coding sequence of *traV* in all three ICEs. Examination of the annotation of *traV* revealed that a much better suited ribosome binding site with a nearly canonical Shine–Dalgarno (SD) sequence is located 75 bp downstream of the original *traV* annotation. As a consequence, we propose to redefine the start codon of *traV* at this new location (Figure 2D and G). Wozniak and Waldor (28) reported that *mosAT*, which is located in HS2 and encodes a toxin-antitoxin system promoting the maintenance of SXT, was induced by SetCD. Clearly, our results show that there is no SetCD-binding site upstream of *mosA*, and that *mosAT* is not differentially expressed upon induction of SetCD by mitomycin C (Supplementary Table S1 and Dataset S1). This suggests that the reported increased expression of *mosAT* likely resulted from read-through of the mRNA transcript initiated by SetCD at the promoter *P_traV_*.

The upstream region of the mutagenic DNA repair system *rumAB* of R391 was reported to contain a strong match to known LexA-binding sites at positions −44 to −25 relative to the *rumA* initiation codon (57). Although we have found a SetCD-binding site 530 bp upstream of *rumA* in the correct orientation to drive *rumA* expression in R391 only (Figure 1B), our RNA-seq data in SXT clearly shows that upon mitomycin C induction, *rumA* is not differentially expressed in a wild-type SXT compared to the corresponding *setCD*-null mutant (Dataset S1). Our data rather support the idea that *rumAB* is part of the LexA regulon, not of the SetCD regulon (57).

The flexibility provided by SetCD has likely helped shaping the complex genetic structure and remarkable plasticity of SXT/R391 ICEs. Ironically, SetCD has also become a beacon signaling the presence of SXT/R391 ICEs to parasitic genomic islands that hijack the ICE transfer machinery. MGIs mimic SetCD-binding sites to activate their own excision in response to the presence of an ICE of the SXT/R391 family in the same cell. We have found here that SetCD binds upstream of *rdfM*, a gene that is known to be activated by SetCD (23), and upstream of *cds4*, a conserved gene of unknown function (Figure 4). Although *int*_MGI_ has been shown to be activated by SetCD (22,23), ChIP-exo failed to identify a statistically significant binding of SetCD upstream of this gene in MGI*Vfl*Ind1 but a more degenerate SetCD motif can be found using the Find Individual Motif Occurrences tool (FIMO) (Figure 4F). This is consistent with our previous report that *int*_MGI_ is induced only 300-fold by SetCD overexpression whereas *rdfM* is induced 2 000-fold in identical conditions (23). Nevertheless, *int*_MGI_ was also shown to be constitutively expressed at low level in the absence of SetCD, thereby allowing integration of the MGI into the chromosome of the recipient cell independently of the cotransfer of an SXT/R391 ICE (23) (Figure 6B). This strategy likely favors MGI's ‘survival’ and dissemination as <2% of recipient cells receiving an MGI have been shown to simultaneously receive a copy of the helper ICE (22). Our data confirm that MGI excision is strictly regulated by the activation of *rdfM* expression, not by *int*_MGI_ overexpression, and is consistent with the inability of an *rdfM*-null mutant to excise and transfer (23).

Unlike in MGIs, we demonstrated here that expression of SXT *int* and *xis* requires the presence of SetCD in both donor and recipient cells. Our results indicate that an SXT Δ*setCD* mutant complemented in *trans* by SetCD only in the donor cells is incapable of integrating site-specifically into the 5′ end of *prfC*. This observation led us to conclude that neither SetCD nor Int are translocated into the recipient strain during conjugation. Instead, *setCD* is expressed *de novo* in the recipient, allowing *int* expression to mediate SXT integration into *prfC* in a site-specific fashion (Figure 6B). This requirement contrasts with the regulation of MGI integration, which is independent of SetCD. As both *int* and *setCD* are physically linked on SXT/R391 ICEs, these mobile elements do not need to rely on a dual promoter regulation of their integrase gene to ensure their survival and dissemination.

Upon entry of an ICE of the SXT/R391 family into a naive host, the repressor SetR is initially absent, and concomitant expression of *setCD* and *setR* likely allows buildup of the SetCD and SetR pools. Entry by conjugation of the ICE as single-stranded DNA is known to activate the SOS response (58), which could transitorily contribute to maintain a low pool of SetR protein, thereby favoring the buildup of SetCD (Figure 6B). However, we showed that the transcript levels are similar for both *xis* and *int* in the presence of SetCD whereas the presence of the RDF Xis in the recipient cells reduces SXT transfer, likely by interfering during the recombination between *attP* and *attB* (25). Therefore a yet uncharacterized mechanism likely maintains a low level of Xis protein or delays its expression in the recipient cell to favor chromosomal ICE integration. In the conjugative transposon Tn*916*, the RDF *xis* and integrase *int* genes are part of a single long tetracycline-inducible mRNA transcript reading through the *attP* attachment site and extending to all of the transfer genes (44). Unlike many mobile integrating elements, *xis* and *int* in SXT/R391 ICEs are not organized as an operon. Instead they are two convergent genes suggesting that other factors besides SetCD regulate their expression.

In summary, our study establishes the SetCD-dependent regulation of excision, transfer and integration the ICEs of the SXT/R931 family as well as the genomic islands they mobilize, and highlights the importance of SetCD in triggering the spread of antibiotic resistance conferring mobile elements in *V. cholerae* populations and in other *Gammaproteobacteria*.

## SUPPLEMENTARY DATA

Supplementary Data are available at NAR Online.

SUPPLEMENTARY DATA
