# Supplementary material for: Transfer activation of SXT/R391 integrative and conjugative elements: unraveling the SetCD regulon
Source: Nucleic Acids Res. 2015 Feb 6;43(4):2045–56. doi: 10.1093/nar/gkv071 (PMC4344509; doi:10.1093/nar/gkv071)
Supplement: SUPPLEMENTARY DATA [file supp_43_4_2045__index.html]

Transfer activation of SXT/R391 integrative and conjugative elements: unraveling the SetCD regulon — Transfer activation of SXT/R391 integrative and conjugative elements: unraveling the SetCD regulon — SUPPLEMENTARY DATA 

# Transfer activation of SXT/R391 integrative and conjugative elements: unraveling the SetCD regulon

## SUPPLEMENTARY DATA

**Files in this Data Supplement:**

- SUPPLEMENTARY DATA
- SUPPLEMENTARY DATA
