# Supplementary material for: Transfer activation of SXT/R391 integrative and conjugative elements: unraveling the SetCD regulon
Source: Nucleic Acids Res. 2015 Feb 6;43(4):2045–56. doi: 10.1093/nar/gkv071 (PMC4344509; doi:10.1093/nar/gkv071)
Supplement: SUPPLEMENTARY DATA [file supp_gkv071_nar-03411-m-2014-File008.pdf]

SUPPLEMENTARY DATA

Text S1 Supplementary Materials and Methods

Bacterial strains

The *E. coli* strains used in this study are described in Table 1. Antibiotics were used at the following concentrations: ampicillin (Ap), 100 µg/ml; chloramphenicol (Cm), 20 µg/ml; erythromycin (Em), 10 µg/ml; spectinomycin (Sp), 50 µg/ml; kanamycin (Kn), 50 µg/ml or 10 µg/ml for single copy integrants of pOPlacZ; nalidixic acid (Nx), 40 µg/ml; sulfamethoxazole (Su), 160 µg/ml; tetracycline (Tc), 12 µg/ml; trimethoprim (Tm), 32 µg/ml; mitomycin C (MC), 100 ng/ml.

Molecular biology methods

Genomic and plasmid DNA were prepared using the Wizard Genomic DNA Purification Kit (Promega) and EZ-10 Spin Column Plasmid DNA Minipreps Kit (Biobasic), respectively, according to manufacturer's instructions. All the enzymes used in this study were purchased from New England BioLabs or Enzymatics. PCR assays were performed with the primers described in Table S2. The PCR conditions were as follows: (i) 3 min at 94˚C; (ii) 30 cycles of 30 sec at 94˚C, 30 sec at the appropriate annealing temperature, and 1 minute/kb at 68˚C; and (iii) 5 min at 68˚C. When necessary, PCR products were purified using a EZ-10 Spin Column PCR Products Purification Kit (Biobasic) according to manufacturer's instructions. *E. coli* was transformed by electroporation according to Dower *et al*. (1). Electroporation was carried out in a BioRad GenePulser Xcell apparatus set at 25 µF, 200 V and 1.8 kV using 1-mm gap electroporation cuvettes. Sequencing reactions were performed by the Plateforme de Séquençage et de Génotypage du Centre de Recherche du CHUL (Québec, QC, Canada).

Plasmid and strain constructions

Plasmids and primers used in this study are described in Table 1 and S2, respectively. The template plasmid pDPL458 was constructed by site-directed mutagenesis of pKD3 to introduce a SetCD box using the primer pair Int-CDbox-F/Int-CDbox-R and the Q5 Site-Directed Mutagenesis Kit (New England Biolabs). The template plasmid pNC12 was constructed by introducing the SPA tag from pJL148 (2) amplified with the primer pair SPACtBamHI.for/SPACtBamHI.rev into the BamHI site of pVI36.

The transcriptional fusion vector pOPlacZ contains the promoterless *E. coli* K12 *lacZ* gene with its native Shine-Dalgarno sequence (3). PCR fragments containing -100, -52 or -35 to +81 with respect to the transcriptional +1 site of SXT *s003*, and -180, -52 or -35 to +11 relative to the +1 of SXT *xis* were cloned between the PstI and XhoI sites of pOPlacZ to produce pDPL440, pDPL382, pDPL465, pDPL384, pDPL385 and pDPL467, respectively.

The Δ*setCD* (DPL3) mutation was introduced in SXT using the one-step chromosomal gene inactivation technique (4) with the primer pair setD2WF/setC2WR and pKD13 as the templates. Similarly, translational fusions of the 3xFLAG tag the C-terminal end of SetC in SXT (DPL492), Orf90 in R391 (DPL491) and SetC in ICE*Vfl*Ind1 (DPL493) were constructed using the same technique with primer pairs DATsetC3xFLAG-F/DATsetC3xFLAG-SXT-R (SXT) or DATsetC3xFLAG-F/DATsetC3xFLAG-R391-R (R391 and ICE*Vfl*Ind1) and pNC12 as the template. The CRP operator upstream of the *lacZYA* operon in *E. coli* MG1655 was substituted by a SetCD box ($lacZo^{CD1}$ and $lacZo^{CD2}$) using the same technique with primer pairs DATcdlacZ-FO3/DATcdlacZ-R3 and DATcdlacZ-FO3/DATcdlacZ-R4, respectively, and pDPL458 as the template, yielding DPL494 and DPL501. The strains DPL453, DPL400, DPL490, DPL393, DPL394 and DPL489 were constructed by transforming *E. coli* BW25113 expressing λ Int from pINT-ts with plasmids pDPL440, pDPL382, pDPL465, pDPL384, pDPL385 and pDPL467, respectively, following the method of Haldimann and Wanner (5). pGG2B was subsequently introduced in these strains to express *setCD* under control of the arabinose-inducible promoter P_{BAD}. All mutations were verified by PCR amplification and sequenced by the Plateforme de séquençage et génotypage des génomes (CHUL, Québec).

β-galactosidase assays

The substrates used to determine the LacZ levels were either o-2-nitrophenyl-β-D-galactopyranoside (ONPG) or 5-bromo-4-chloro-3-indolyl-β-D-galactopyranoside (X-gal). The β-galactosidase assays using ONPG were carried out as described previously (6) in LB medium (for strains DPL453, DPL400, DPL490, DPL393, DPL394, and DPL 489) or in M9 minimal medium supplemented with 1% glycerol and 1% thiamine (for strains MG1655, DPL494, and DPL501), and 100 µg/ml ampicillin for maintenance of pGG2B. The induction of *setCD* expression, the alleviation of LacI repression, and catabolic repression were done by supplementing with 0.02% arabinose, 1 mM isopropyl-β-D-thiogalactopyranoside (IPTG), or 0.2% glucose, respectively, to a refreshed culture grown to an OD_{600nm} of 0.2 followed by 2-h incubation at 37°C with shaking prior cell sampling. For the assays using X-gal as a substrate, a single colony of each strain was re-suspended in 50 µl of LB broth by vortexing and streaked onto M9 minimal medium supplemented with 1.5% agar, 20 mg/ml X-gal, 1% glycerol, 1% thiamine and 100 mg/ml ampicillin for maintenance of either pBAD30 or pGG2B. Derepression or activation of the P_{lac} derivatives was done by further supplementing the media with 0.02% arabinose, 0.2% glucose and 1 mM IPTG.

Southern blot hybridization

The fragment *attP* for Southern blot hybridization was amplified by PCR using genomic DNA of HW220 as a template and primers CattPF/CAttPR (Table S2) (7). Chromosomal DNA was prepared using the Wizard Genomic DNA purification kit (Promega) as described in the

manufacturer's instructions, followed by a phenol:chloroform:isoamyl alcohol (25:24:1) extraction and ethanol precipitation. Purified total genomic DNA was digested with EcoRI and EcoRV restriction enzymes, and analyzed by Southern blot hybridization (8) using the 602-bp EcoRI fragment of pDPL189 containing *attP* from SXT as a probe. The probe was radiolabeled using T4 polynucleotide kinase and dATP (γ-^{32}P) 3000 Ci/mmol and purified with an illustra MicroSpin G-25 column (GE Healthcare). The purified radiolabeled probe was added to the prehybridization solution (6X SSC, 5X Denhardt's reagent, 0.5% (w/v) SDS and 100 µg/ml sonicated salmon sperm DNA) with a specific activity of 2×10^{6} cpm/µg. Radioactive signal was detected using a Storm 860 Molecular Imager (GMI) and brightness and contrasts were adjusted on the entire image using Quantity One 1-D analysis software (Bio-Rad).

CHEF-PFGE

E. coli chromosomal DNA used for contour-clamped homogeneous electric field pulsed field gel electrophoresis (CHEF-PFGE) was prepared as previously described (9). After digestion by the SpeI restriction enzyme, large *E. coli* restriction fragments were separated with a CHEF-DR II system (Bio-Rad) using the conditions described in Burrus and Waldor (10).

Real-Time quantivative PCR analysis

Total RNAs for reverse transcription real-time quantitative PCR (qRT-PCR) were extracted from *E. coli* strains HW220, DPL3, AD72, AD132 and AD133, and cDNA were synthesized as described elsewhere (11). The RNA quality was further verified with a 2100 Bioanalyzer instrument (Agilent Technologies). qRT-PCR was performed by measuring the increase of fluorescence using the Quantifast SYBR Green mix (QIAGEN) in an Eppendorf RealPlex (Eppendorf). Quantitative amplification of *int*, *xis* and *cds4* was carried out using primer pairs RTint-F/RTint-R, RTxis-F/RTxis-R and RTgene4Vf-F/RTgene4Vf-R respectively. For normalization, *rpoZ*, the gene coding for ω subunit of the RNA polymerase, was amplified using primers RTrpoZcoli-F and RTrpoZcoli-R and results were expressed as a relative expression based on the ΔCt calculation method. Experiments were carried out three times in triplicate and combined. Melting curves were carried out on the final reaction products (156-165 bp) to verify that amplification was specific to targets. All primer pairs exhibited efficiencies comprised between 91% and 94%. The detection limit was fixed at 0.0004, the mean of all results obtained with SXT Δ*setCD*.

Primer extension analysis

Primer extension experiment to determine the transcription start site of *s003* and *rdfM* was carried out as described elsewhere (12) using total RNA extracted respectively from HW220 and AD72 induced with 100 ng/ml mitomycin C. The primers used for the primer extension reactions using the Primer Extension System-AMV Reverse Transcriptase kit (Promega) were RRintR (*s003*) and

PE-rdfM-2 (*rdfM*) (Table S2). A Sanger sequencing ladder was generated using the Sequenase 2.0 DNA Sequencing Kit (Affymetrix-USB).

SetCD ChIP-exo experiments and ChIP-exo libraries preparation

Chromatin immunoprecipitation coupled with exonuclease digestion (ChIP-exo) experiments and ChIP-exo libraries preparation were performed as described previously (3) with the following modifications. ChIP-exo experiments were performed in biological triplicates, each using 10 ml of *E. coli* strains DPL491, DPL492, or DPL493 bearing $setC^{3xFLAG}$ ($orf90^{3xFLAG}$ for R391) grown to an OD_{600nm} of ~0.6 with agitation in an orbital shaker at 37°C. Cultures were then induced with 200 ng/ml mitomycin C and incubated for an additional 2 hours at 37°C. Induced cultures were next subjected to 1% formaldehyde crosslinking for 20 min at 25°C followed by quenching with 0.125 M glycine. Immunoprecipitation procedure was then performed as described previously (3) using the Anti-FLAG M2 antibody (Sigma). ChIP-exo libraries were prepared as described previously (3) using the following primers (Table S2): TrueSeq-B-dT24-VN, 3'-T-IGA-A0 adaptor (IGA-A0-up annealed with IGA-A0-down-T/A; 30 nM adaptor final concentration), IGA-PCR-PE-F, and TrueSeq-MPEX-R with a specific barcode for each sample. The resulting libraries were purified with Agencourt AMPure XP SPRI beads (Beckman Coulter) with a ratio of 1.0, before evaluating the library size and concentration using a 2100 Bioanalyzer instrument (Agilent Technologies). Barcoded libraries were pooled together in equal quantity to obtain approximately the same amount of reads per sample (Table S3).

Total RNA extractions and RNA-seq libraries preparation

Total RNA extractions were done with three biological replicates. 10 ml of *E. coli* DPL492 (SXT $setC^{3xFLAG}$) and 5 ml of *E. coli* DPL3 (SXT Δ*setCD*) were grown to an OD_{600nm} of ~0.3 as described for ChIP-exo experiments. DPL492 culture was split in two, and one half was induced with 200 ng/ml mitomycin C for two hours along with the entire DPL3 culture. All cultures reached a final OD_{600nm} of ~0.6 and total RNA was then extracted using the Direct-zol RNA MiniPrep kit (Zymo Research) according to the manufacturer's specifications. Purified total RNA was treated with 1.5 U of DNAse I in 1X DNAse I Reaction Buffer (Zymo Research) at 37°C for 20 min. DNAse-treated RNA samples were then purified using the RNA Clean & Concentrator-5 kit (Zymo Research) according to the manufacturer's specifications. The quality and concentration of total RNA extractions were evaluated using a 2100 Bioanalyzer instrument (Agilent Technologies). A total of nine RNA sequencing (RNA-seq) libraries were constructed in biological triplicates per experimental condition as described previously (3) using the following primers (Table S2): TrueSeq-3'-hybrid-B0, 5'-hybrid-A0, DSN-TrueSeq-F, DSN-TrueSeq-R, IGA-PCR-PE-F, and TrueSeq-MPEX-R with a specific barcode for each sample. Indexed libraries were pooled together in equal quantity to obtain approximately the same amount of reads per sample (Table S3).

Genome-wide 5'-RACE RNA libraries preparation

Equal quantities of purified total RNA from non-induced DPL492, induced DPL492, and induced DPL3 replicates were pooled to obtain a final amount of 2 µg for each experimental condition. 5' rapid amplification of cDNA ends (5'-RACE RNA) libraries were then prepared as described previously (3) using the following primers (Table S2) : TrueSeq-3'-hybrid-B0, 5'-hybrid-A0, IGA-PCR-PE-F, and TrueSeq-MPEX-R with a specific barcode for each sample. The resulting libraries were purified with Agencourt AMPure XP SPRI beads (Beckman Coulter) with a ratio of 0.9, before evaluating the library size and concentration using a 2100 Bioanalyzer instrument (Agilent Technologies). Indexed libraries were pooled together in equal quantity to obtain approximately the same amount of reads per sample (Table S3).

Illumina sequencing and data analysis

Illumina sequencing was performed on a Illumina HiSeq 2000 Sequencing system at the Plateau de biologie moléculaire et génomique fonctionnelle of the Institut de Recherche Cliniques de Montréal. Approximately 3 to 6 million paired-end reads of 50 bp were obtained for each library, each represented in triplicates in RNA-seq and ChIP-exo experiments (Table S3). Sample multiplexing relied on a 6-bp barcode located within the TrueSeq-MPEX-R primer (Table S2).

ChIP-exo data analysis

Only the forward reads of the Illumina paired-end reads obtained from SetCD ChIP-exo experiments (Table S3) were aligned using the Burrows-Wheeler Aligner (BWA) (13) since reverse reads do not contain interesting information using this type of library (3). The reference genomes used for the alignments are *E. coli* MG1655 genome (GenBank: U00096.3) as well as the appropriate ICE sequence: SXT (strain DPL492), GenBank: AY055428.1; R391 (strain DPL491), GenBank: AY090559.1; and ICE*Vfl*Ind1/MGI*Vfl*Ind1 (strain DPL493), GenBank: KM213605.1 (ICE*Vfl*Ind1) along with KC117176.1 (MGI*Vfl*Ind1). Alignment from biological triplicates were filtered, combined and sorted as described elsewhere (3). Normalized read density (Fig. 1B and Fig. 4B) as well as peak enrichments were calculated by the Model-based Analysis of ChIP-Seq tool (MACS) (14) as described elsewhere (3). Subpeaks (referred as "peaks" in the paper; Fig. 1B, Fig. 4B and Table S1) with a summit height below 40X of the theoretical bp coverage (obtained by calculating the ratio between the number of reads used by MACS and the combined *E. coli* MG1655 and respective ICE/MGI genome size) were discarded to eliminate the background signal. SetCD-binding logos (Fig. 1D) were created separately for each ICE independently by providing to the motif-based sequence analysis tool (MEME) (15) the nucleotide sequences corresponding to subpeaks summits ±100 bp with the "any number of repetitions" option and a maximum motif length of 20 bp (note that only the subpeaks found within the ICE sequences were provided to MEME for motifs elicitation). Precise locations of SetCD-

binding motifs within each ICE were determined using the Motif Alignment and Search Tool (MAST) (16) and their respective matrix from MEME, and intersected with MACS subpeaks. The MAST option to process the negative DNA strand as a separated sequence was used, and only motifs with *p*-value below 1.0e-04 positioned completely within MACS peaks were considered significant hits (Fig. 1B and Fig. 2). If two or more motifs in the same orientation were found within one peak, only the most significant *p*-value was conserved. The combined SetCD-binding logo was generated by submitting to MEME all significant SetCD motif sequences found by MAST in all three ICEs with the "One motif per sequence" option (Fig. 1D). Positions of SetCD binding motifs inside MGI*Vfl*Ind1 were identified by MAST with the ICE*Vfl*Ind1 SetCD binding-site matrix using the same parameters as for motifs searching in ICEs (Fig. 4B, 4C, and 4F). The degenerate SetCD-binding motifs located within the intergenic regions upstream *traI* were identified by providing the SetCD-binding matrices found for each ICE respectively to the Find Individual Motif Occurrences tool (FIMO) (17). Only motifs correctly oriented relative to the downstream gene with a *p*-value below 1.0e-03 were considered significant (Fig. S3). The degenerate SetCD-binding motif located within the intergenic region upstream int_{MGI} was identified by the same method using the ICE*Vfl*Ind1 SetCD binding-site matrix. High-resolution ChIP-exo data was generated by processing the filtered and sorted BWA aligned sequences to conserve only the first nucleotide of forward reads. Finally, near-single nucleotide resolution ChIP-exo reads density (Fig. 2A-F, 4C and S3A), as well as full reads ChIP-exo density, were calculated with Bedtools' genomecov function (18) for each DNA strand seperately.

RNA-seq data analysis

Paired-end Illumina reads obtained from RNA-seq libraries (Table S3) were trimmed and filtered as described elsewhere (3). Filtered reads were then aligned on the *E. coli* MG1655 genome (GenBank U00096.3) as well as the SXT sequence (GenBank AY055428.1) using the Estimated Degree of Gene Expression in Prokaryotes software (EDGE-pro) (19), which also generated reads per kilobase of transcript per million mapped reads values (RPKM) for each gene (Fig. 1C and Dataset S1). Differential expression analysis between RNA-seq experimental conditions (Dataset S1 and Table S3) was then conducted using the DESeq R package (20) with the following options to calculate data dispersion: pooled method, fit-only sharing mode, and local fitType. Statistically, genes between two given conditions were considered differentially expressed if the calculated *p*-value was equal or below 0.1 and the expression fold change was equal or above 5 (Dataset S1). To generate strand-specific RNA-seq density files for data visualization using a UCSC trackhub, filtered reads were also aligned by BWA on the *E. coli* MG1655 genome (GenBank U00096.3) as well as SXT (GenBank AY055428.1). Aligned reads were filtered with SAMtools view to discard those with an alignment quality score below 10, and combined per experimental condition using SAMtools merge. Pooled as well as individual filtered

reads alignments were then sorted with SAMtools. RNA-seq reads density calculated per dataset with Bedtools genomecov for each DNA strand separately.

Genome-wide 5'-RACE data analysis and SetCD-regulated promoters characterization

Forward reads of Illumina paired-end sequences, which correspond to the start of transcribed RNAs, obtained from the 5'-RACE libraries (Table S3) were trimmed, aligned by BWA, filtered and sorted by SAMtools as described elsewhere (3). The reads were then processed to conserve only the first nucleotide, corresponding to potential transcription start sites. Single-bp resolution 5'-RACE reads density was calculated with Bedtools genomecov for each DNA strand separately (Fig. 1C). Properly oriented transcription start sites from the mitomycin C induced wild-type SXT that were located less than 125 bp downstream of a SetCD-binding motif were considered significant and reported in the SetCD-regulated promoter alignment (Fig. 2G).

SUPPLEMENTARY REFERENCES

1. Dower, W.J., Miller, J.F. and Ragsdale, C.W. (1988) High efficiency transformation of *E. coli* by high voltage electroporation. *Nucleic Acids Res*, **16**, 6127-6145.

2. Zeghouf, M., Li, J., Butland, G., Borkowska, A., Canadien, V., Richards, D., Beattie, B., Emili, A. and Greenblatt, J.F. (2004) Sequential Peptide Affinity (SPA) system for the identification of mammalian and bacterial protein complexes. *J. Proteome Res.*, **3**, 463-468.

3. Carraro, N., Matteau, D., Luo, P., Rodrigue, S. and Burrus, V. (2014) The Master Activator of IncA/C Conjugative Plasmids Stimulates Genomic Islands and Multidrug Resistance Dissemination. *PLoS Genet.*, **10**, e1004714.

4. Datsenko, K.A. and Wanner, B.L. (2000) One-step inactivation of chromosomal genes in *Escherichia coli* K-12 using PCR products. *Proc. Natl. Acad. Sci. U. S. A.*, **97**, 6640-6645.

5. Haldimann, A. and Wanner, B.L. (2001) Conditional-Replication, Integration, Excision, and Retrieval Plasmid-Host Systems for Gene Structure-Function Studies of Bacteria. *J. Bacteriol.*, **183**, 6384-6393.

6. Miller, J.F. (1992) *A short course in bacterial genetics*. Cold Spring Harbor Laboratory Press, Plainview, NY.

7. Burrus, V. and Waldor, M.K. (2003) Control of SXT integration and excision. *J. Bacteriol.*, **185**, 5045-5054.

8. Sambrook, J., Fritsch, E. and Maniatis, T. (1989) *Molecular Cloning: A laboratory Manual*. Cold Spring Harbor Laboratory Press, Cold Spring Harbor, NY.

9. Heath, J.D., Perkins, J.D., Sharma, B. and Weinstock, G.M. (1992) NotI genomic cleavage map of *Escherichia coli* K-12 strain MG1655. *J. Bacteriol.*, **174**, 558-567.

10. Burrus, V. and Waldor, M.K. (2004) Formation of SXT tandem arrays and SXT-R391 hybrids. *J. Bacteriol.*, **186**, 2636-2645.

11. Daccord, A., Mursell, M., Poulin-Laprade, D. and Burrus, V. (2012) Dynamics of the SetCD-Regulated Integration and Excision of Genomic Islands Mobilized by Integrating Conjugative Elements of the SXT/R391 Family. *J. Bacteriol.*, **194**, 5794-5802.

12. Garriss, G., Poulin-Laprade, D. and Burrus, V. (2013) DNA-Damaging Agents Induce the RecA-Independent Homologous Recombination Functions of Integrating Conjugative Elements of the SXT/R391 Family. *J. Bacteriol.*, **195**, 1991-2003.

13. Li, H. and Durbin, R. (2009) Fast and accurate short read alignment with Burrows-Wheeler transform. *Bioinformatics*, **25**, 1754-1760.

14. Zhang, Y., Liu, T., Meyer, C.A., Eeckhoute, J., Johnson, D.S., Bernstein, B.E., Nusbaum, C., Myers, R.M., Brown, M., Li, W. *et al.* (2008) Model-based analysis of ChIP-Seq (MACS). *Genome Biol*, **9**, R137.

15. Bailey, T.L. and Elkan, C. (1994) Fitting a mixture model by expectation maximization to discover motifs in biopolymers. *Proc Int Conf Intell Syst Mol Biol*, **2**, 28-36.

16. Bailey, T.L. and Gribskov, M. (1998) Combining evidence using p-values: application to sequence homology searches. *Bioinformatics*, **14**, 48-54.

17. Grant, C.E., Bailey, T.L. and Noble, W.S. (2011) FIMO: scanning for occurrences of a given motif. *Bioinformatics*, **27**, 1017-1018.

18. Quinlan, A.R. and Hall, I.M. (2010) BEDTools: a flexible suite of utilities for comparing genomic features. *Bioinformatics*, **26**, 841-842.

19. Magoc, T., Wood, D. and Salzberg, S.L. (2013) EDGE-pro: Estimated Degree of Gene Expression in Prokaryotic Genomes. *Evol Bioinform Online*, **9**, 127-136.

20. Anders, S. and Huber, W. (2010) Differential expression analysis for sequence count data. *Genome Biol*, **11**, R106.

Table S1. Detailed description of SetCD ChIP-exo enrichment peaks and SetCD-binding motifs for all three ICEs and MGI*Vfl*Ind1.

ICE or MGI	MACS ChIP peak calls				SetCD-binding motifs			
	**Shared**	**Peak position**	**Summit**	**Peak height**	**Position**	**Orientation**	***p*-value**	**Oriented**
SXT	I	251-1311	615	12017	547-566	Positive	2.9E-10	*xis*
	II	3001-4181	3425	16467	3450-3469	Negative	1.9E-08	*s003*
					3470-3489	Positive	4.9E-08	*mobI*
	-	20851-21580	21150	2930	-	-	-	-
	III	23901-25131	24485	6683	-	-	-	-
	-	-	-	-	45100-45119[a]	Positive[a]	6.9E-07[a]	*traI*
	IV	46801-47711	47165	6615	47112-47131	Positive	6.3E-08	*traD*
	V	50641-51651	51155	2807	51103-51122	Positive	7.4E-09	*traL*
	VI	53911-55451	54335	15008	54243-54262	Negative	1.0E-06	-
					54263-54282	Positive	1.4E-08	*traV*
	VII	56951-57701	57305	3826	57284-57303	Positive	4.4E-08	*dsbC*
	VIII	71311-72581	71735	28440	71666-71685	Negative	2.0E-07	*s063*
					71686-71705	Positive	2.4E-07	*s089*
	IX	88291-89121	88745	8118	88695-88714	Positive	9.3E-09	*traF*
R391	I	2261-2751	2525	5772	2457-2476	Positive	1.5E-09	*xis*
	II	4991-6441	5455	13262	5360-5379	Negative	2.1E-08	*s003*
					5380-5399	Positive	2.6E-08	*mobI*
	III	7881-8291	8105	4496	-	-	-	-
		8301-8971	8495	3410	8409-8428	Negative	1.3E-06	*rumA*
	-	-	-	-	32275-32294[a]	Positive[a]	9.5E-07[a]	*traI*
	IV	34171-34891	34435	4033	34287-34306	Positive	8.8E-05	*traD*
	V	37901-39661	38295	4569	38376-38395	Positive	2.1E-09	*traL*
	VI	41211-42671	41695	15622	41536-41555	Positive	2.0E-07	*traV*
		42681-43221	42795	3211	-	-	-	-
	VII	49121-50141	49565	3062	49489-49508	Negative	1.1E-06	*cds55*
					49509-49528	Positive	6.4E-08	*dsbC*
	VIII	60491-62331	61465	27717	61366-61385	Negative	8.9E-07	*s063*
					61386-61405	Positive	8.1E-09	*s089*
	IX	74121-74881	74325	7398	74271-74290	Negative	1.6E-06	-
					74291-74310	Positive	3.7E-09	*traF*
ICE*Vfl*Ind1	I	71-1180	455	12511	404-423	Positive	4.1E-10	*xis*
	II	5311-6081	5565	12265	5577-5596	Negative	3.9E-08	*s003*
					5597-5616	Positive	6.5E-08	*mobI*
	III	25781-27201	26585	4534	-	-	-	-
	-	42781-43361	43115	2627	43063-43082[a]	Positive[a]	4.8E-04[a]	*traI*
					43338-43357	Positive	9.3E-06	
	IV	44901-45860	45115	3791	45074-45093	Positive	6.0E-08	*traD*
	V	52911-53771	53500	2674	53438-53457	Positive	2.3E-09	*traL*
	VI	56281-57711	56655	8473	56598-56617	Positive	3.0E-08	*traV*
	VII	59381-59901	59680	1944	59616-59635	Positive	6.5E-08	*dsbC*
	VIII	71021-72250	71495	20690	71473-71492	Negative	4.7E-07	*s063*
					71493-71512	Positive	9.4E-09	*s089*
	IX	91581-92650	92155	5248	92076-92095	Negative	4.0E-07	*s075*
					92096-92115	Positive	4.3E-09	*traF*
MGI*Vfl*Ind1	-	-	-	-	229-248[b]	Positive[b]	1.4E-04[b]	int_{MGI}
	-	4991-6771	5685	11440	5653-5672	Positive	7.0E-05	*cds4*
	-	17531-19201	18335	8408	17958-17977	Positive	1.6E-05	*cds15*
					18318-18337	Negative	9.3E-06	*rdfM*

[a] Motifs obtained by FIMO using the corresponding ICE SetCD-binding logo depicted in Fig. 1D.
[b] Motif obtained by FIMO using the ICE*Vfl*Ind1 SetCD-binding logo depicted in Fig. 1D.

Table S2. DNA sequences of the primers used in this study.

Primer name	Nucleotide sequence (5' to 3')	Use in the study
Int-CDboxF	CGAACAGGCAGATAGATGAAAGGATTTGTCACCTTTCTGCCCACC	Cloning of the SetCD box of *P_{s003}*
Int-CDboxR	CGGGTGGGCAGAAAGGTGACAAATCCTTTCATCTATCTGCCTGTT	Cloning of the SetCD box of *P_{s003}*
DATcdlacZ-FO3	GACTGGAAAGCGGGCAGTGAGCGCAACGCAATTAATCATATGAATATCCTCCTTAG	Replacement of the CRP box and -35 element of *P_{lac}* promoter by the SetCD box of *P_{s003}*
DATcdlacZ-R3	ACAATTCCACACAACATACGAGCCGGAAGCATAAAGTGTAAAGGCAGAAAGGTGACAA	Replacement of the CRP box of *P_{lac}* promoter by the SetCD box of *P_{s003}*
DATcdlacZ-R4	ACAATTCCACACAACATACGAGCCGGAAGCATAAAGGCGGTGGGCAGAAAGGTGACAA	Replacement of the CRP box and -35 of *P_{lac}* promoter by the SetCD box and -35 of *P_{s003}*
DATsetC3xflag-F	ATGGCCGAGCAAACCACTAGAGCACTTTTTGAGAACGGTGAGCTCGACTACAAAGA	C-terminal translational fusion of SPA tag with *setC* in SXT, R391 and ICE*Vfl*Ind1
DATsetC3xflag-SXT-R	GCACGGGCGGTGCACAATCAAATCATGTATCAGCATGTGTAGGCTGGAGCTGCTTC	C-terminal translational fusion of SPA tag with *setC* in SXT
DATsetC3xflag-R391-R	GCACGGGCGGTGCACAATCAAATCATGCATCAGCATGTGTAGGCTGGAGCTGCTTC	C-terminal translational fusion of SPA tag with *setC* in R391 and ICE*Vfl*Ind1
SPACtBamHI.for	GGGATCCTCCATGGAAAAGAGAAGATGGA	Construction of pNC12
SPACtBamHI.rev	GGGATCCCTACTTGTCATCGTCATCCTTG	Construction of pNC12
RRintR	ACGACGTTTGGCGTCTCGAT	Identification +1 of *s003*
PE-rdfM-2	TGGATGATTGTGTAGTTGGGT	Identification of +1 of *rdfM*
RTrpoZcoli-F	GCTCGTCAGATGCAGGTAGG	Amplification of *rpoZ*
RTrpoZcoli-R	GCTTGTAATTCAGCGGCTTC	Amplification of *rpoZ*
RTint-F	AGTTTTATGACTGGCTGATT	Amplification of *int*
RTint-R	TTTTTGACTCATGCAAATAG	Amplification of *int*
RTxis-F	TAACCCCTTTTCTAACATCT	Amplification of *xis*
RTxis-R	TTCTCATACTCCTCCAAATC	Amplification of *xis*
RTgene4Vf-F	GCAATGGCCAAACCTGTATT	Amplification of *cds4*
RTgene4Vf-R	GGCGTAATGCACCAAAAGAT	Amplification of *cds4*
CattPF	TTCGAAGGTTTAGCCACAGTTGTTTATGAGTG	Amplification of *attP*$_{SXT}$
CattPR	TTCGAATATTCCGCTTTTGTAATGTCGAAA	Amplification of *attP*$_{SXT}$
setD2WF	CTATCGCAATATTTTACATGAACTGGAGGTCCGTCAATTCCGGGGATCCGTCGACC	Deletion of *setCD* in SXT
setC2WR	CACGGGCGGTGCACAATCAAATCATGTATCAGCATGGTGTAGGCTGGAGCTGCTTCG	Deletion of *setCD* in SXT
VISRF	CTCTCATTAACTGGGTTCAGG	Amplification of *attR*
VISLR3	GCATTCTCCTGAAAATCAATG	Amplification of *attL*
EattBF	GCCGCACTTTTGCCATTATT	Amplification of *attB* and *attL*
prfCQR	TTGTTGGAGATTGGACGGAAC	Amplification of *attB*
EattBR	AGCAGCACCTTCTCGGTGAT	Amplification of *attR*
TrueSeq-B-dT24-VN[a]	AGACGTGTGCTCTTCCGATCTTTTTTTTTTTTTTTTTTTTTTTTVN	Second strand synthesis in ChIP-exo libraries preparation
IGA-A0-up[b,c]	/5AmMC6/ACACTCTTTCCCTACACGACGCTCTTCCGATCT	ChIP-exo libraries preparation
IGA-A0-down-T/A[c]	GATCGGAAGAGCGTCGTGTAGGGAAAGAGTGTAGAT	ChIP-exo libraries preparation
IGA-PCR-PE-F	AATGATACGGCGACCACCGAGATCTACACTCTTTCCCTACACGACGCTCTTCCGATCT	ChIP-exo and RNA-seq libraries amplification
TrueSeq-MPEX-R[d]	CAAGCAGAAGACGGCATACGAGAT-index-GTGACTGGAGTTCAGACGTGTGCTCTTCCGATC	ChIP-exo and RNA-seq libraries amplification, contains 6 bp barcode
TrueSeq-3'-hybrid-B0[b,e]	/5Phos/rArGrArUrCrGrGrArArGAGCACACGTCT/3AmMO/	RNA-seq libraries preparation
5'-hybrid-A0[e]	ACACGACGrCrUrCrUrUrCrCrGrArUrCrU	RNA-seq libraries preparation
DSN-TrueSeq-F	CACGACGCTCTTCCGATCT	RNA-seq libraries amplification
DSN-TrueSeq-R	AGACGTGTGCTCTTCCGATCT	RNA-seq libraries amplification

[a] Mixed bases: V: A, C or G; N: A, T, C or G.
[b] Nucleotide modifications: /5AmMC6/ : 5' amino modifier C6; /5Phos/ : 5' phosphorylation; /3AmMO/ : 3' amino modifier.
[c] Form the 3'-T-IGA-A0 adaptor when annealed together.
[d] Index corresponds to an unique combination of 6 nucleotides and serve as a barcode for sample multiplexing.
[e] r letter corresponds to RNA bases.

Table S3. Illumina libraries sequenced in this study.

Sequencing line	Library type	Experimental condition	Replicate	Index in TrueSeq-MPEX-R oligo	Index read by instrument	Sequenced paired-end reads (millions)
1	ChIP-exo	DPL492 (SXT *setC*^{3xFLAG}) +MC	1	AGTACC	GGTACT	3.1
			2	CAGACC	GGTCTG	3.0
			3	GCAACC	GGTTGC	2.8
		DPL491 (R391 *orf90*^{3xFLAG}) +MC	1	CCTTAC	GTAAGG	3.2
			2	GGCGAC	GTCGCC	4.2
			3	ATAGAC	GTCTAT	3.0
		DPL493 (ICE*Vfl*Ind1 *setC*^{3xFLAG} MGI*Vfl*Ind1) +MC	1	GATAAC	GTTATC	3.1
			2	ACGAAC	GTTCGT	3.3
			3	CTCAAC	GTTGAG	3.1
2	RNA-seq	DPL492 (SXT *setC*^{3xFLAG})	1	TGACCG	CGGTCA	4.5
			2	CCGCTC	GAGCGG	5.0
			3	GAGGTC	GACCTC	5.4
		DPL492 (SXT *setC*^{3xFLAG}) +MC	1	AGCCGC	GCGGCT	4.0
			2	CGGTTG	CAACCG	4.5
			3	GTAATT	AATTAC	4.9
		DPL3 (SXT Δ*setCD*) +MC	1	ATGACT	AGTCAT	5.3
			2	TCCAGC	GCTGGA	6.0
			3	CCTACT	AGTAGG	5.4
	5'-RACE	DPL492 (SXT *setC*^{3xFLAG})	1	GTTGAT	ATCAAC	9.0
		DPL492 (SXT *setC*^{3xFLAG}) +MC	1	GCGGCT	AGCCGC	7.1
		DPL3 (SXT Δ*setCD*) +MC	1	ACTCTT	AAGAGT	6.9

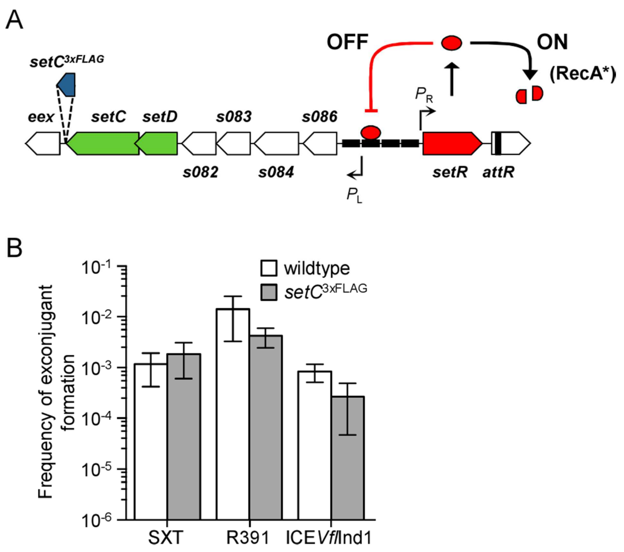

Figure S1. The bimodal switch governing the conjugative transfer of SXT/R391 ICEs and MGIs. (A) Schematic representation of the regulatory region of SXT/R931 ICEs. The fate of SetR proteins (red ellipse) governs the state of transfer (ON/OFF). Genes are represented by arrows and are color coded as follow: red, transcriptional repressor SetR; green, transcriptional activators SetC and SetD; white, other or unknown functions. 3xFLAG-labelling of the C-terminal coding sequence of *setC* is indicated in blue. (B) SetC^{3xFLAG} remains functional to activate the conjugative transfer of SXT/R391 ICEs. Conjugation assays using *E. coli* VB17 (BW25113 SXT), DPL513 (BW25113 R391), AD57 (CAG18439 ICE*Vfl*Ind1 MGI*Vfl*Ind1), and their *setC*^{3xFLAG} derivatives (DPL492, DPL491 and DPL493, respectively) as donors upon mitomycin C induction. The recipient strains were either *E. coli* CAG18439 or VB111 (MG1655 Nx^{r}). Results are the means and standard deviations of three independent biological replicates.

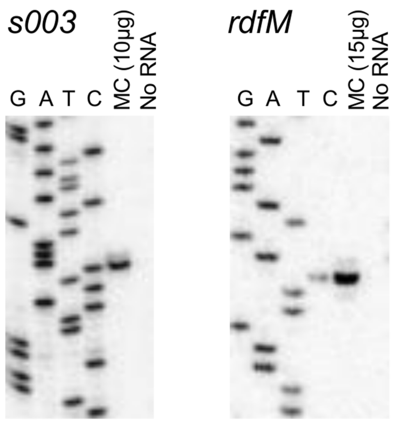

Figure S2. Primer extension analysis of the promoters of *s003* in SXT and *rdfM* in MGI*Vfl*Ind1. G, A, T and C, Sanger sequencing reaction lanes; MC, mitomycin C-induced samples; No RNA, negative control. Concentrations of RNA used for the primer extension reaction are indicated. Each well was loaded with equal reaction volumes.

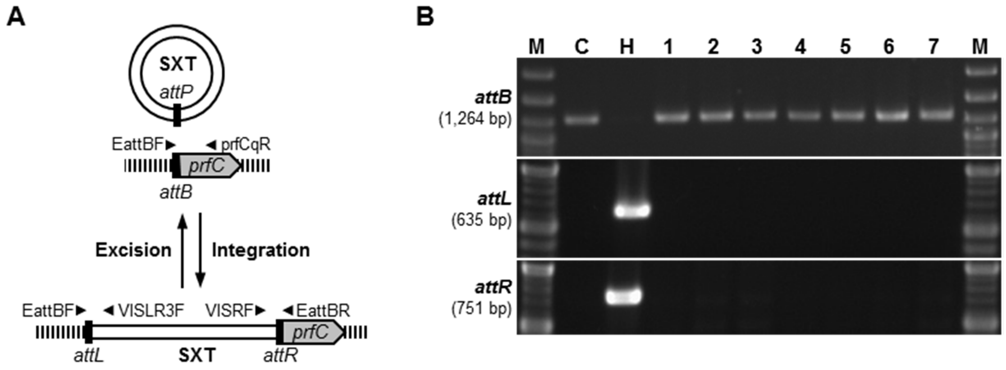

Figure S3. Atypical maintenance of SXT Δ*setCD* in fresh exconjugants. (A) Model of SXT integrating into and excising from the 5' end of *prfC* in a site-specific fashion. (B) PCR amplification of *attB*, *attL* and *attR* junction fragments. Ethidium bromide-stained 1% agarose gels of PCR products amplified using primer pairs EattBF/prfCqR (*attB*), EattBF/VISLR3 (*attL*) and VISRF/EattBR (*attR*) (Table S2). The position of the primers is indicated in panel A. Lanes: M, 2-Log DNA Ladder molecular size marker; C, CAG18439 (SXT- strain, negative control); H, HW220 (CAG18439 containing SXT integrated into the 5' end of *prfC*); 1 to 7, Nx^r Su^r Tm^r exconjugants resulting from a mating experiment between *E. coli* DPL5 and *E. coli* VB111 (mating *e* in Figure 5A).

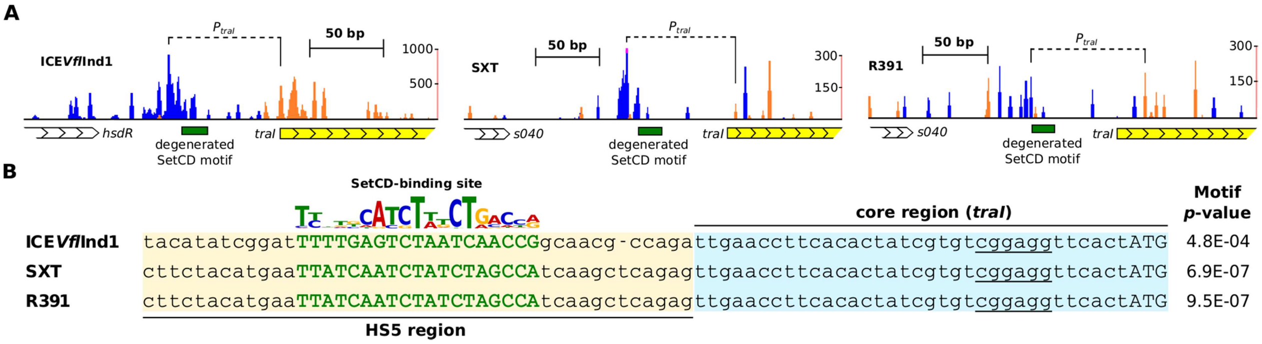

Figure S4 The *traI* promoter is disrupted by the insertion of variable DNA. (A) ChIP-exo binding profile of SetCD in the intergenic region upstream of *traI* in ICE*Vfl*Ind1, SXT and R391, respectively. Tracks are organized as in Figure 2A-F. The green boxes correspond to degenerate SetCD binding-motifs located on the positive DNA strand that were detected within ChIP-exo peaks by FIMO but not by MAST analysis. Genes are represented by arrows and are color coded as in Figure 1. (B) Alignment of the *traI* upstream sequences showing the proposed SetCD-binding motifs in all three ICEs. The blue shaded sequences correspond to identical sequences shared by the three ICEs (core). The orange shaded sequences correspond to variable DNA (HS5) that is shared by SXT and R391 but different in ICE*Vfl*Ind1. The degenerate SetCD-binding motifs found in panel A are represented in bold green capital letters with their respective *p*-value. The combined SetCD logo is as in Figure 1D. Shine-Dalgarno sequences are underlined while start codons are in capital letters.
